# Targeted Use of Sustainable Aviation Fuel to Maximize Climate Benefits

**DOI:** 10.1021/acs.est.2c05781

**Published:** 2022-11-17

**Authors:** Roger Teoh, Ulrich Schumann, Christiane Voigt, Tobias Schripp, Marc Shapiro, Zebediah Engberg, Jarlath Molloy, George Koudis, Marc E. J. Stettler

**Affiliations:** † Department of Civil and Environmental Engineering, Imperial College London, LondonSW7 2AZ, U.K.; ‡ Institute of Atmospheric Physics, Deutsches Zentrum für Luft- und Raumfahrt, 82234Oberpfaffenhofen, Germany; § Institute of Atmospheric Physics, University Mainz, 55099Mainz, Germany; ∥ Institute of Combustion Technology, Deutsches Zentrum für Luft- und Raumfahrt, 70569Stuttgart, Germany; ⊥ Orca Sciences, 4110 Carillon Point, Kirkland, Washington98033, United States; # NATS, 4000 Parkway, Whiteley Fareham, HampshirePO15 7FL, U.K.

**Keywords:** aviation, contrail cirrus, climate forcing, sustainable aviation fuels, mitigation

## Abstract

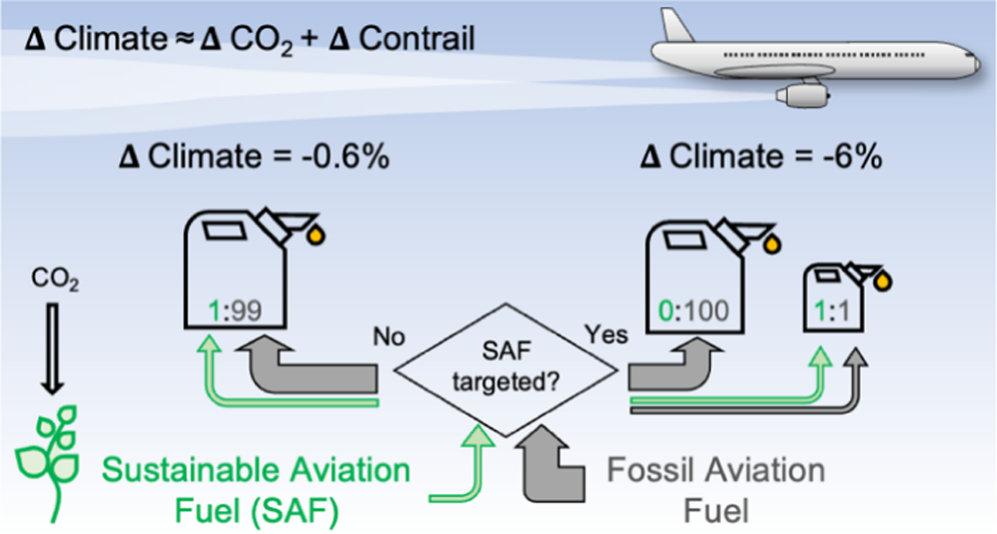

Sustainable aviation fuel (SAF) can reduce aviation’s CO_2_ and non-CO_2_ impacts. We quantify the change in contrail properties and climate forcing in the North Atlantic resulting from different blending ratios of SAF and demonstrate that intelligently allocating the limited SAF supply could multiply its overall climate benefit by factors of 9–15. A fleetwide adoption of 100% SAF increases contrail occurrence (+5%), but lower nonvolatile particle emissions (−52%) reduce the annual mean contrail net radiative forcing (−44%), adding to climate gains from reduced life cycle CO_2_ emissions. However, in the short term, SAF supply will be constrained. SAF blended at a 1% ratio and uniformly distributed to all transatlantic flights would reduce both the annual contrail energy forcing (EF_contrail_) and the total energy forcing (EF_total_, contrails + change in CO_2_ life cycle emissions) by ∼0.6%. Instead, targeting the same quantity of SAF at a 50% blend ratio to ∼2% of flights responsible for the most highly warming contrails reduces EF_contrail_ and EF_total_ by ∼10 and ∼6%, respectively. Acknowledging forecasting uncertainties, SAF blended at lower ratios (10%) and distributed to more flights (∼9%) still reduces EF_contrail_ (∼5%) and EF_total_ (∼3%). Both strategies deploy SAF on flights with engine particle emissions exceeding 10^12^ m^–1^, at night-time, and in winter.

## Abstract 2

Climate benefits of sustainable aviation fuel could be 9−15 times higher if targeted onto flights with the most warming contrails.

## 1 Introduction

Aviation emissions consist of both CO_2_ and non-CO_2_ components, and their relative contribution to anthropogenic climate forcing is expected to increase due to air travel demand growth and limited potential for rapid decarbonization.^1−4^ The use of sustainable aviation fuel (SAF) is considered as one of the solutions^5−100^ to reach the aviation industry’s commitment of achieving net zero CO_2_ emissions by 2050.^9^ The International Civil Aviation Organization (ICAO) defines SAF as renewable or waste-derived fuel that meets several sustainability criteria,^10^ including but not limited to: (i) the reduction in net life cycle greenhouse gas emissions by at least 10% relative to conventional fuels; (ii) not being produced from biomass in lands with high carbon stocks; and (iii) conserving the local water, soil, air quality, and food security. As of January 2022, seven different SAF production pathways have been certified^11,12^ to be blended with conventional kerosene at up to a 50% blending ratio by volume (*p*_blend_). The Fischer–Tropsch synthetic paraffinic kerosene (FT-SPK) was the first pathway approved in 2009, while the use of hydroprocessed esters and fatty acid SPK (HEFA-SPK) is the most mature pathway that is in commercial use.^11,12^

Recent studies^13,14^ estimated that the life cycle well-to-wake (WTW) CO_2_-equivalent (CO_2_e) emissions of SAF range from 5.2 to 73.4 gCO_2_e MJ^–1^, depending on feedstock, technology pathways, and energy source, and thus can be up to 94% lower than the WTW emissions from conventional fuel (88.9 gCO_2_e MJ^–1^). While the CO_2_ life cycle benefits are significant, SAF only accounted for 0.01% of the global jet fuel use in 2018,^15^ and its supply is only projected to increase to ∼2% of the global jet fuel demand in 2025.^16^ An increase in SAF supply that is comparable to the production growth in ethanol and biodiesel in the early 2000s, translating to ∼60 new bio-refineries per annum (p.a.), could reduce aviation CO_2_e emissions by 15% in 2050 relative to the baseline scenario with conventional fuels.^17^ Without supply bottlenecks, aviation CO_2_ emissions could be reduced by 5.5–9.5% over 15 years if the adoption rate of SAF increases by 1–2% p.a..^6^

In addition to the CO_2_ benefits, SAF can also reduce the nonvolatile particulate matter (nvPM) number emissions index (EI*_n_*) by up to 70%^18−23^ relative to conventional fuels, with the reduction in nvPM EI*_n_* varying as a function of engine thrust settings, fuel hydrogen, and aromatic content.^18,19^ nvPM emissions at cruise altitudes contribute to contrail formation when conditions in the exhaust plume satisfy the Schmidt–Appleman criterion (SAC).^24−26^ In the soot-rich regime (EI*_n_* > 10^13^ kg^–1^), the nvPM EI*_n_* is positively correlated with the initial contrail ice crystal number and optical depth (τ_contrail_) and negatively correlated with the ice crystal size.^24,27^ Indeed, recent in situ measurements of young contrail properties^23,28^ found that replacing conventional jet fuel with SAF led to significant differences in the ice number concentration (up to −70%), ice crystal size (+40%), and τ_contrail_ (−52%), and these changes are expected to reduce the contrail lifetime and climate forcing.^29−33^ However, several studies^34,35^ estimate that SAF could increase the contrail occurrence by 1–8% because its water vapor emissions index (EI_H_2_O_) can be up to 10% higher than that of conventional fuels.^24,34,36^ While the effects of SAF on contrail occurrence and changes to contrail properties have been measured, the effects of a lower nvPM EI*_n_* on the contrail cirrus net radiative forcing (RF) have so far only been quantified with modeling studies: Schumann et al.^33^ computed a 39% reduction in global annual mean contrail cirrus net RF for a 50% reduction in nvPM EI*_n_*; Bock & Burkhardt^37^ and Burkhardt et al.^31^ found 15 and 50% reduction in the global contrail cirrus net RF, respectively, when SAF is used across the fleet; and Caiazzo et al.^35^ reported a −4 to +18% change in the contrail net RF over the United States.

To mitigate aviation’s CO_2_ impact, the European Commission aims to impose a mandate that requires aviation fuel supplies at European Union (EU) airports to be blended with SAF.^38^ From 2025 onward, the regulation proposes a minimum *p*_blend_ of 2%, and gradually increasing to 85% by 2050.^38,39^ Yet it is unclear how the SAF will be distributed. In 2019, only 39 out of 1657 EU airports accounted for 80% of conventional fuel used by flights departing EU airports, and there may be logistical benefits to focusing the SAF supply chain on specific airports.^40^ Our hypothesis is that if SAF were targeted to flights that are forecast to form strongly warming contrails, a higher overall climate benefit could be realized. For example, a recent study^41^ has found that transatlantic flights with strongly warming contrails are more common during the winter, at dusk, above low-level water clouds, and for specific aircraft types with high nvPM number emissions.

This paper aims to: (i) extend an existing methodology^18^ to estimate the changes in nvPM EI*_n_* from SAF with different *p*_blend_ values; (ii) quantify the change in contrail occurrence, properties, and climate forcing in the North Atlantic when SAF is adopted by the fleet at different blend ratios; and (iii) evaluate the potential to maximize the overall climate benefits of SAF when the limited supply is deployed to flights that would otherwise form strongly warming contrails.

## 2 Materials and Methods

The dataset and methods used in this study include: (i) an air traffic dataset for the North Atlantic provided by the U.K. air navigation service provider (NATS), containing the actual trajectory from 477,923 flights that traversed the Shanwick and Gander Oceanic Area Control Centre in 2019; (ii) meteorology from the European Centre for Medium-Range Weather Forecast (ECMWF) ERA5 high-resolution realization (HRES) reanalysis^42^ (0.25° × 0.25° horizontal resolution for 37 pressure levels and at a 1 h temporal resolution) with corrections applied to the humidity fields^41^ so the probability density function is consistent with in situ observations;^43,44^ (iii) the Base of Aircraft Data Family 4.2 (BADA 4) and Family 3.15 (BADA 3) models from EUROCONTROL;^45,46^ (iv) the ICAO Aircraft Emissions Databank (EDB);^47^ and (v) the contrail cirrus prediction model (CoCiP).^29,30^ These datasets and methods have been documented in Teoh et al.^41^ Here, we focus on the methodologies used to estimate the changes in aircraft nvPM EI*_n_* and fuel properties from SAF with different *p*_blend_ values. Further details not included in the main text are in the Supporting Information.

### 2.1 Aircraft Performance and Emissions

The aircraft types covered by BADA 4 account for 91.5% of flights in the air traffic dataset, while BADA 3 is available for all flights. As BADA 4 provides more accurate aircraft performance estimates across the whole operational flight envelope relative to BADA 3,^48^ it is selected as the preferred method to estimate the fuel mass flow rate (*ṁ*_f_) and overall propulsion efficiency (η). For each flight, we assume^41^ that the aircraft mass at the first waypoint is equal to the nominal (reference) mass provided by BADA, and the mass decreases over subsequent waypoints in line with the fuel consumption.

The aircraft-engine combinations are identified from BADA, and where possible, the engine-specific data from the ICAO EDB^47^ is used to estimate the nvPM EI*_n_* at each waypoint. As of July 2021, the ICAO EDB^47^ contains nvPM EI*_n_* data for 47 identified aircraft-engine pairs, and we use the measurements that have been corrected for dilution, thermophoretic, and particle line losses.^41,49^ For aircraft types with nvPM measurements included in the ICAO EDB (68.6% of all flights), the nvPM EI*_n_* is estimated by linear interpolation relative to the nondimensional engine thrust settings which captures the unique emissions profile from different combustor types.^41^ For aircraft types in which nvPM measurements are not covered by the ICAO EDB (31.1% of flights), we use the fractal aggregates model,^32,50,51^ which estimates the nvPM EI*_n_* using model estimates of the mass emissions index,^52,53^ particle size distribution, and morphology based on the emissions profile of single annular combustors. For the remaining flights where engine-specific data is not available, a constant nvPM EI*_n_* of 10^15^ kg^–1^ is assumed. We note that these nvPM estimates are for conventional fuels with a hydrogen mass content (H_fuel_) of 13.8%,^47^ and adjustments must be made to account for the effects of SAF.

### 2.2 Change in nvPM and Fuel Properties due to SAF

Two approaches are available to estimate the change in nvPM EI_n_ from different *H*_fuel_ (Brem et al.^18^ and the ICAO CAEP/11 model,^54^ described in the Supporting Information S1). However, Brem et al.^18^ is only valid for engine thrust settings (*F̂*) above 30% and for cases where the arithmetic difference in H_fuel_ between the reference fuel and SAF (Δ*H*) is below 0.6%, and extrapolating beyond these bounds can lead to unrealistic values where ΔnvPM EI*_n_* ≤ 100% (Figure S1); while the ICAO CAEP/11 model^54^ can only be applied within an allowable H_fuel_ range of 13.4–14.3%.

Here, we extend the methodology of Brem et al.^18^ using the latest measurements from the NASA ACCESS^22^ and ECLIF2/ND-MAX^21,23^ campaigns, which investigated the SAF effects on nvPM EI*_n_* under a wider range of engine thrust settings (10% < *F̂* < 100%) and higher Δ*H* (up to 1.1%). A piecewise function retains the original formulation at low Δ*H* (≤ 0.5%), and an exponential term is added when Δ*H* > 0.5% to ensure that the estimated ΔnvPM EI*_n_* asymptotically approaches −100%
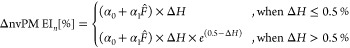
1where α_0_ = −114.21 and α_1_ = 1.06 are the original coefficients from Brem et al.^18^*F̂* is approximated by dividing the *ṁ*_f_ at mean sea level (MSL) conditions (*ṁ*_f_^MSL^) by the maximum *ṁ*_f_ (*ṁ*_f,max_^MSL^) provided by the ICAO EDB.^47^ For cruise conditions, *ṁ*_f_^Cruise^ is converted to an equivalent *ṁ*_f_^MSL^ using the Fuel Flow Method 2 (FFM2) methodology^55^
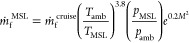
2where *T*_amb_ and *p*_amb_ are the ambient temperature and pressure, respectively; *T*_MSL_ (288.15 K) and *p*_MSL_ (101325 Pa) are the standard atmospheric temperature and pressure at MSL, respectively; and *M* is the Mach number. Equation 1, also visualized in Figure S3, is evaluated by comparison to ground and cruise measurements from four experimental campaigns:^18,19,21−23^ the coefficient of determination (*R*^2^) and normalized mean bias (NMB) for the measured and estimated ΔnvPM EI*_n_* are, respectively, 0.84 and +28% when compared against ground measurements, and 0.83 and −3.2% against cruise measurements (Figure S5).

Data from different experimental campaigns show that the fuel properties are generally linear relative to the SAF *p*_blend_, including: (i) H_SAF_, which is required to compute Δ*H*; (ii) lower calorific value (LCV), which influences η and the SAC threshold temperature;^24^ and (iii) EI_H_2_O_ (Figure S7). Therefore, a linear interpolation is used to estimate these quantities for different *p*_blend_. We assume that the reduction in CO_2_ from SAF arises from the difference in WTW life cycle emissions that is between 10 and 94% lower than conventional fuels: the lower bound (−10%) represents the minimum reduction in CO_2_ WTW life cycle emissions that is required for a fuel to be certified as SAF;^10^ while the upper bound (94%) represents the SAF production pathway with the lowest CO_2_ WTW life cycle emission (5.2 gCO_2_e MJ^–1^, FT-SPK produced from municipal solid waste).^13^ The CO_2_ energy forcing (EF), which describes the cumulative climate forcing of CO_2_ over a selected time horizon, is calculated to approximate the CO_2_ climate benefits from SAF^32,50^
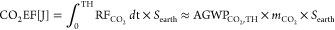
3where AGWP_CO_2_,TH_ is the CO_2_ absolute global warming potential (2.92 × 10^–6^ sW m^–2^ kg^–1^-CO_2_ for a 100-year time horizon),^56^*m*_CO_2__ is the total CO_2_ emissions, and *S*_Earth_ is Earth’s surface area (5.101 × 10^14^ m^2^).^57^

### 2.3 Contrail Simulation

CoCiP simulates the life cycle of each contrail segment formed along an individual flight trajectory.^30^ A contrail segment is formed when two consecutive waypoints satisfy the SAC, and the initial contrail ice crystal number depends on the: (i) nvPM EI*_n_*, where a lower bound is set at 10^13^ kg^–1^ to account for ambient aerosols and organic particles;^27^ (ii) *T*_amb_ influencing the nvPM activation rate;^26^ and (iii) fraction of ice particles that survive the wake vortex phase.^30^ Persistent contrail segments, i.e., contrail segments that survive the wake vortex phase, are then simulated with model time-steps of 1800 s until their end of life, defined as when the contrail ice crystal number falls below the background ice nuclei concentration (<10^3^ m^–3^), τ_contrail_ decreases to below 10^–6^, or when the lifetime exceeds a maximum of 24 h.^30^ For each waypoint, CoCiP computes the local contrail radiative forcing (RF′), the change in radiative flux over the contrail area,^29^ and the RF′ for each contrail segment is aggregated to estimate the annual mean contrail cirrus net RF over the North Atlantic. The contrail energy forcing (EF_contrail_), calculated as the product of the contrail segment RF′, length, and width and integrated over the lifetime of the contrail segment, represents the cumulative climate forcing for each contrail segment that can then be aggregated for a specific flight.^32,41,58,59^

### 2.4 SAF Scenarios

The emissions and simulated contrail outputs for the baseline scenario with conventional fuels were published in Teoh et al..^41^ In this paper, six additional simulations were performed by assuming a fleetwide adoption of SAF with different *p*_blend_, ranging from 1% to 100% (Table 1). We note that the stated H_fuel_ for a given *p*_blend_ in Table 1 assumes the use of conventional fuel with a 13.8% H_fuel_, and variabilities in the composition of the conventional fuel and SAF can lead to differences in H_fuel_ for a given *p*_blend_ for other use cases (Supporting Information S2).To account for real-world supply constraints, we assume that the available SAF supply is equal to 1% of the total fuel consumption in 2019 (8.9 × 10^7^ kg) and evaluate strategies to maximize the overall climate benefits of SAF. The limited supply can either be: (i) uniformly distributed to all flights with a 1% blend ratio; or blended at higher ratios and targeted to (ii) flights with the largest EF_contrail_ in the baseline simulation; or (iii) flights with the largest absolute reduction in EF_contrail_ between the baseline and SAF simulations (ΔEF_contrail_).

**Table 1:** Summary of the Simulation Runs and the Assumed Fuel Properties That are Used in This Study, Where Contrails are Simulated with Conventional Kerosene and SAF with Different Homogeneous Blending Ratios

simulation	blending ratio (*p*_blend_) (%)	H_fuel_ (%)	Δ*H* (%)	LCV (MJ kg^–1^)	EI_H_2_O_(kg kg^–1^)
Baseline	0	13.80	0	43.10	1.237
SAF1	1	13.815	0.015	43.11	1.238
SAF10	10	13.95	0.150	43.21	1.250
SAF30	30	14.25	0.450	43.42	1.277
SAF50	50	14.55	0.750	43.64	1.304
SAF70	70	14.85	1.050	43.85	1.331
SAF100	100	15.30	1.500	44.17	1.371

## 3 Results and Discussion

### 3.1 Fleetwide Adoption of SAF

**Figure 1:**
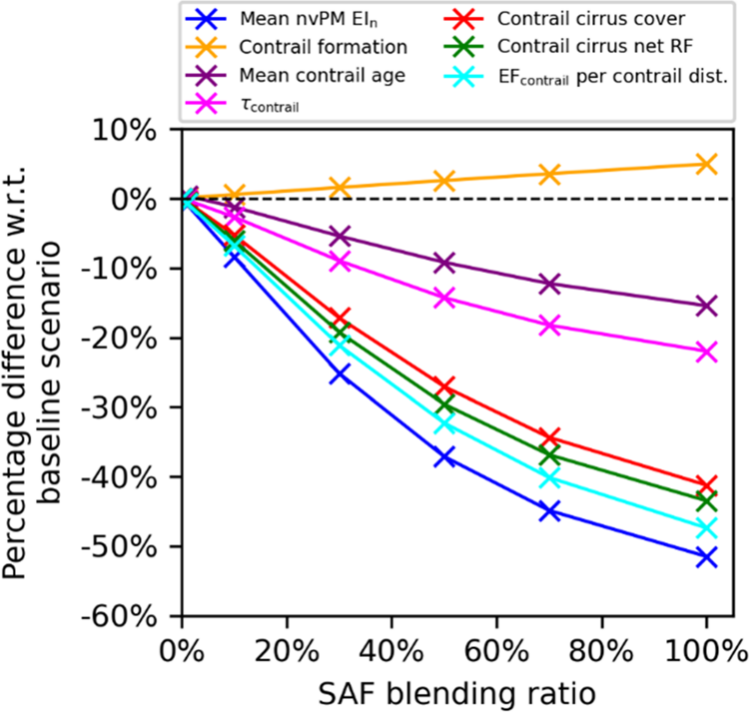
Relative difference in the fleet-aggregated nvPM EI*_n_*, contrail properties, and climate forcing in the North Atlantic for different homogeneous SAF blending ratios relative to the baseline scenario where conventional fuels are used.

Table 2 summarizes the fleet-aggregated CO_2_ and nvPM emissions, contrail occurrence, properties, and climate forcing for the different simulation runs. Figure 1 shows the change in simulated contrail properties relative to the baseline scenario. These estimates are also compared with existing studies^31,33,35,37^ that directly and indirectly modeled the effects of SAF on contrails in the Supporting Information S3.3.

**Table 2:** Fleet-Aggregated Fuel Consumption, nvPM Emissions, and Contrail Statistics in the North Atlantic for 2019, Where Flights are Powered by Conventional Kerosene Fuel (Baseline), and SAF with Different Blending Ratios

	2019 North Atlantic							
fleet-aggregated emissions and contrail properties	baseline^a^	SAF1	SAF10	SAF30	SAF50	SAF70	SAF100	(%) change: SAF100 vs baseline
total fuel burn (×10^9^ kg)	8.922	8.920	8.903	8.865	8.828	8.791	8.736	–2.1
fuel burn per distance (kg km^–1^)	7.538	7.536	7.522	7.490	7.459	7.428	7.381	–2.1
total CO_2_ emissions (×10^9^ kg)^c^	28.2	27.9/28.2	25.5/27.8	20.2/27.2	14.8/26.5	9.53/25.8	1.66/24.8	–94.1/–11.9
CO_2_ EF (×10^18^ J)^c^	42.0	41.6/41.9	38.0/41.5	30.1/40.5	22.1/39.5	14.2/38.5	2.47/37.0	–94.1/–11.9
mean nvPM EI*_n_*(×10^15^ kg^–1^)	0.94	0.93	0.86	0.70	0.59	0.52	0.46	–51.5
flights forming persistent contrails (%)	54.58	54.60	54.70	54.89	55.08	55.25	55.49	1.7
flight distance forming persistent contrails (%)	16.21	16.22	16.30	16.47	16.63	16.79	17.01	5.0
persistent contrail distance (×10^8^ km)	1.919	1.920	1.929	1.949	1.968	1.987	2.014	5.0
lifetime-mean ice particle number per contrail length (*n*_ice_) (×10^12^ km^–1^)	3.19	3.14	2.89	2.32	1.92	1.65	1.43	–55.1
lifetime-mean ice particle volume-mean radius (*r*_ice_) (μm)	7.24	7.30	7.47	7.92	8.35	8.71	9.09	25.5
mean contrail age (*h*)	3.52	3.53	3.47	3.33	3.19	3.09	2.97	–15.4
contrail optical depth (τ_contrail_)	0.122	0.121	0.118	0.111	0.104	0.099	0.095	–22.0
contrail cirrus coverage with (τ_contrail_ > 0.1) (%)	0.473	0.471	0.448	0.392	0.345	0.311	0.278	–41.2
number of flights: warming contrails	208,965	209,083	209,781	211,516	212,913	214,067	215,473	3.1
number of flights: cooling contrails	51,889	51,880	51,620	50,829	50,321	49,975	49,717	–4.2
proportion of flights with warming contrails (%)	80.11	80.12	80.3	80.6	80.9	81.1	81.3	1.4
mean SW RF′ (W m^–2^)	–3.220	–3.210	–3.134	–2.936	–2.768	–2.641	–2.519	–21.8
mean LW RF′ (W m^–2^)	4.647	4.637	4.560	4.357	4.174	4.032	3.890	–16.3
mean net RF′ (W m^–2^)^b^	1.4271	1.4266	1.4263	1.4201	1.4065	1.3918	1.3715	–3.9
annual mean SW RF (mW m^–2^)	–236	–235	–221	–187	–161	–143	–126	–46.5
annual mean LW RF (mW m^–2^)	471	469	442	377	327	291	259	–45.0
annual mean net RF (mW m^–2^)	235	234	221	190	166	149	133	–43.5
EF_contrail_(×10^18^ J)	62.7	62.4	58.8	50.3	43.6	38.9	34.6	–44.8
EF_contrail_ per flight distance (×10^8^ J m^–1^)	0.53	0.53	0.50	0.42	0.37	0.33	0.29	–44.8
EF_contrail_ per contrail length (×10^8^ J m^–1^)	3.27	3.25	3.05	2.58	2.21	1.96	1.72	–47.4
EF_total_: CO_2_ + contrails(×10^18^ J)^c^	104.7	103.9/104.3	96.8/100.3	80.3/90.7	65.7/83.0	53.1/77.4	37.1/71.6	–64.6/–31.6

a Results for the baseline simulation, where flights are powered by conventional kerosene fuel, are obtained in Teoh et al.^41^

b Five significant figures to allow for the identification of differences in values.

c The two values arise from assumptions on the lower and upper bound of the CO_2_ life cycle emissions from SAF.

#### 3.1.1 Emissions and Contrail Properties

A fleetwide adoption of fully synthetic SAF leads to a reduction in the: (i) total fuel consumption (−2.1%, when comparing SAF100 versus the baseline scenario) because of the higher fuel LCV (+2.5%); (ii) total CO_2_ emissions (between −12 and −94%, depending on assumptions on the reduction in CO_2_ WTW emissions from SAF); and (iii) mean nvPM EI*_n_* (−51%) because of a higher H_fuel_ (+11%). We note that the mean nvPM EI*_n_* for all SAF simulations are in the “soot-rich” regime, exceeding 10^13^ kg^–1^ by more than an order of magnitude (Table 1 and Supporting Information S3.1), and therefore, organic volatile particles and ambient natural aerosols are unlikely to activate and form contrail ice crystals.^27^

Comparing the baseline scenario and SAF100, the total persistent contrail length increases by 5% and a higher proportion of flights form persistent contrails (55.5% of all flights) vs the baseline scenario (54.6%) due to the higher EI_H_2_O_. Around 267,000 flights formed persistent contrails in the baseline scenario, and for 69% of these contrail-forming flights, the change in persistent contrail length exhibits a power law distribution (Figure S9a), ranging from +13 to +163 km (5th–95th percentile) with a median of +28 km. Furthermore, additional contrails are generally formed at the edges of ISSRs where RHi ≈ 100% and the higher EI_H_2_O_ pushes the conditions over the threshold for contrail persistence (Figure S10).

Both the larger EI_H_2_O_ and lower mean nvPM EI*_n_* from SAF100 contribute to a 25% increase in mean ice particle volume-mean radius (*r*_ice_) over the contrail life cycle as the larger amount of condensable water in the exhaust is distributed across a smaller number of condensation nuclei.^60^ This, in turn, shortens the mean contrail lifetime by 15% because it increases the sedimentation rate and reduces the time required for ice crystals to encounter subsaturated layers of the atmosphere.^27,58^ The shorter contrail lifetime (−15%) offsets the small increase in the persistent contrail formation (+5%), thereby reducing the annual mean contrail cirrus coverage by up to 41% (0.47% coverage in the baseline simulation vs 0.28% in SAF100, shown in Table 2 and Figure S11).

CoCiP estimates τ_contrail_ to be proportional to the number of contrail ice crystal per contrail length (*n*_ice_), the square of *r*_ice_, and the contrail effective depth (i.e., the plume cross-sectional area divided by its width).^30^ Although the change in *r*_ice_ (+25%) is expected to produce larger τ_contrail_ values, the reduction in *n*_ice_ (−55%) and contrail lifetime (−15%), which lowers the contrail segment effective depth, dominates, and causes the τ_contrail_ in SAF100 to be 22% smaller than in the baseline simulation (Figure S11).

#### 3.1.2 Climate Forcing

SAF causes the proportion of flights with warming contrails (EF_contrail_ > 0) to increase from 80.1% (baseline) to 81.3% (SAF100) (Table 2). This is likely due to a smaller τ_contrail_ (up to −22%), which impacts the mean contrail SW RF′ (−22%) more strongly^29^ than the LW RF′ (−16%), leading to a small absolute reduction in the mean contrail net RF′ (−3.9%). However, reductions in the annual mean contrail cirrus net RF (−44%) and EF_contrail_ per contrail distance (−47%) are significantly larger than the mean contrail net RF′ (−3.9%) because of the smaller lifetime (−15%) and coverage area (−41%) (Table 2).

**Figure 2:**
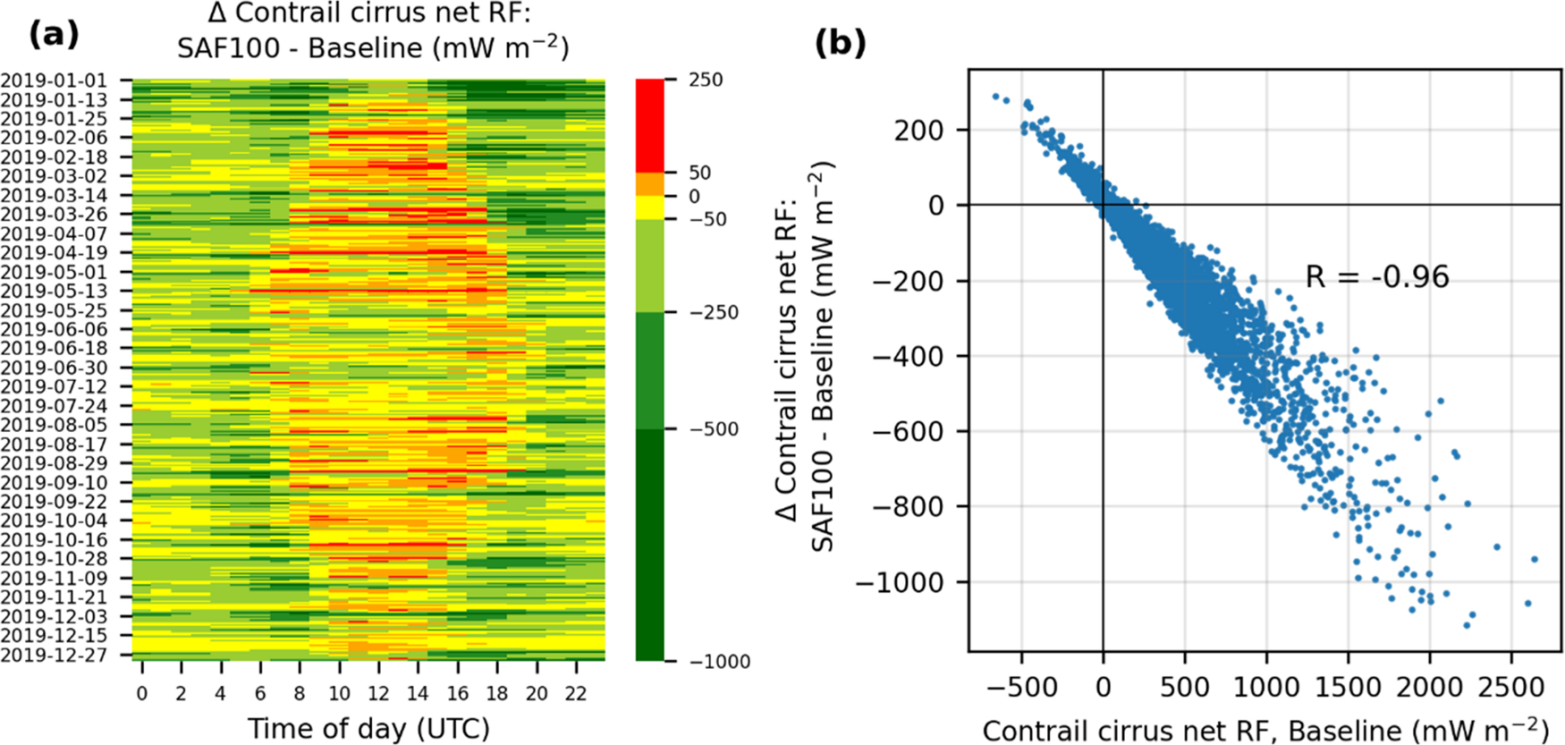
Effectiveness of SAF in reducing the contrail cirrus net RF in the North Atlantic by: (a) time of day (*x*-axis) and day of year (*y*-axis), where the color bar denotes the difference in contrail cirrus net RF between SAF100 vs the baseline simulation with conventional fuels, and (b) relative to the baseline contrail cirrus net RF for each hour in 2019. The baseline contrail cirrus net RF for each hour in 2019 is presented in Figure 3a of Teoh et al.^41^

The change in contrail cirrus net RF exhibits a diurnal dependence (Figure 2a). During the night (solar direct radiation, SDR = 0), SAF reduces the hourly mean contrail net RF by 45% (from 293 in the baseline scenario to 162 mW m^–2^ in SAF100). This is because a smaller τ_contrail_ reduces the LW RF′ while the SW RF′ is already at zero. In daylight hours, SAF also reduces the hourly mean contrail net RF by −43% (from 220 to 126 mW m^–2^), on average. However, for 20% of the hourly time periods (Figure 2a) and for 28% of all contrail-forming flights (Figure S9b), SAF increases the contrail climate forcing because a lower τ_contrail_ reduces the SW RF′ more strongly than the LW RF′.^29^Figure 2b shows that the mitigation potential of SAF increases with the magnitude of hourly contrail cirrus net RF in the baseline simulation, suggesting that a fleetwide adoption of SAF might not be the most optimal solution in the case of limited SAF availability.

We also estimate a 12 to 94% reduction in the annual CO_2_ EF from SAF, which arise from the reduction in total fuel consumption (up to −2.1%) and CO_2_ WTW life cycle emissions (between −10 and −94%). When the reduction in annual EF_contrail_ is included (up to −45%), reductions in the total energy forcing (EF_total_, arising from contrails, total fuel consumption, and the change in CO_2_ WTW life cycle emissions) due to SAF ranges from 32 to 65% (Table 2).

### 3.2 Targeted Use of SAF

While a fleetwide adoption of fully synthetic SAF can significantly reduce the contrail climate forcing in the North Atlantic, it is not feasible because the quantity of SAF is severely constrained in the near term.^15^ Given that ∼12% of all flights over the North Atlantic are responsible for 80% of the annual EF_contrail_ in 2019,^41^ a strategy that deploys the limited supply to flights that would form strongly warming contrails (Section 2.4), mainly at night and in winter (Figure 2a), could maximize the overall climate benefits of SAF and minimize the unintended consequences of increasing the contrail net warming effect.

**Figure 3:**
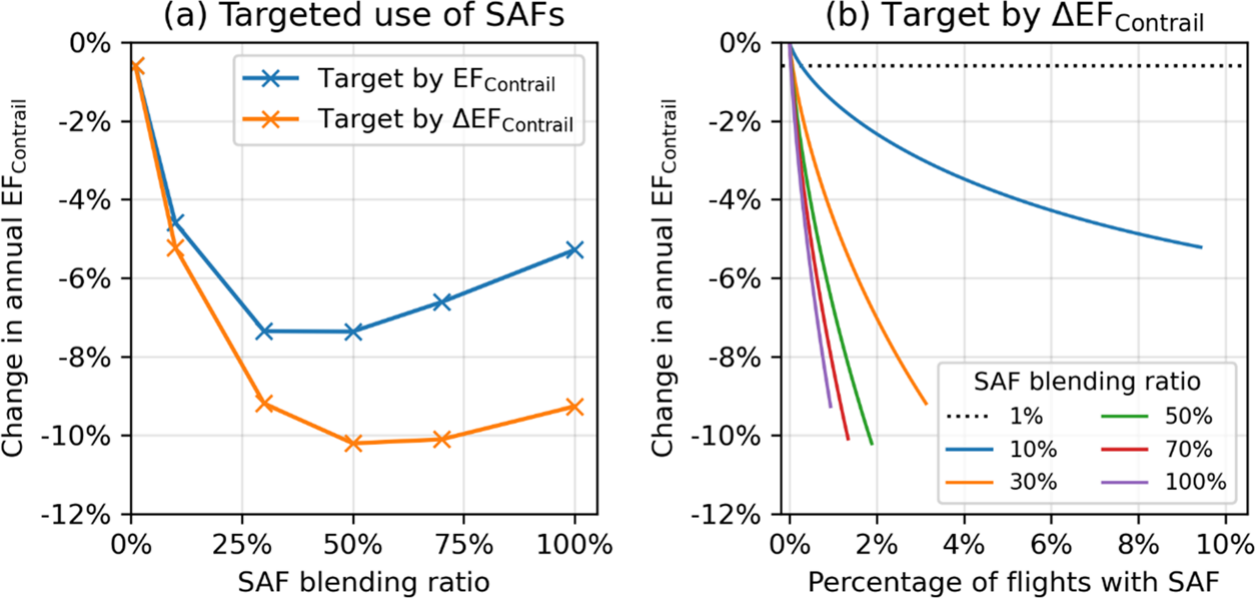
Change in the annual EF_contrail_ in the North Atlantic as a function of (a) SAF blending ratio that is provided to flights with the largest EF_contrail_ (blue line) and ΔEF_contrail_ (orange line) and (b) the percentage of flights that is targeted with SAF from the different blending ratios. Detailed data tables can be found in Supporting Information S4 (Table S8).

A uniform distribution of SAF with a 1% blend (SAF1) reduces the annual EF_contrail_ in the North Atlantic by ∼0.6% relative to the baseline (Table 2). However, the same supply could achieve significantly larger reductions in the annual EF_contrail_ when blended at higher ratios, which induces a larger reduction in the nvPM EI*_n_*, and allocated to flights by order of their EF_contrail_ (up to −7%) or ΔEF_contrail_ (−10%) (Figure 3a). The maximum reduction in annual EF_contrail_ (−10%) is achieved with a 50% *p*_blend_ and targeted to ∼1.9% of flights with the largest ΔEF_contrail_. Further increases in *p*_blend_ beyond 50%, which further concentrates the limited supply to fewer flights, yields a smaller reduction in the annual EF_contrail_ relative to the distribution with 50% *p*_blend_ (Figure 3 and Table S8). Although SAF provided at a 10% *p*_blend_ approximately halves the contrail mitigation potential (∼5% reduction in the annual EF_contrail_ vs ∼10% for *p*_blend_ = 50%), it might be considered as a “low-risk” strategy because SAF is distributed more widely (9.4% of all flights vs. 1.9% for *p*_blend_ = 50%), thereby accounting for uncertainties in forecasting the subset of flights with the largest ΔEF_contrail_ (Figure 3b).

**Figure 4:**
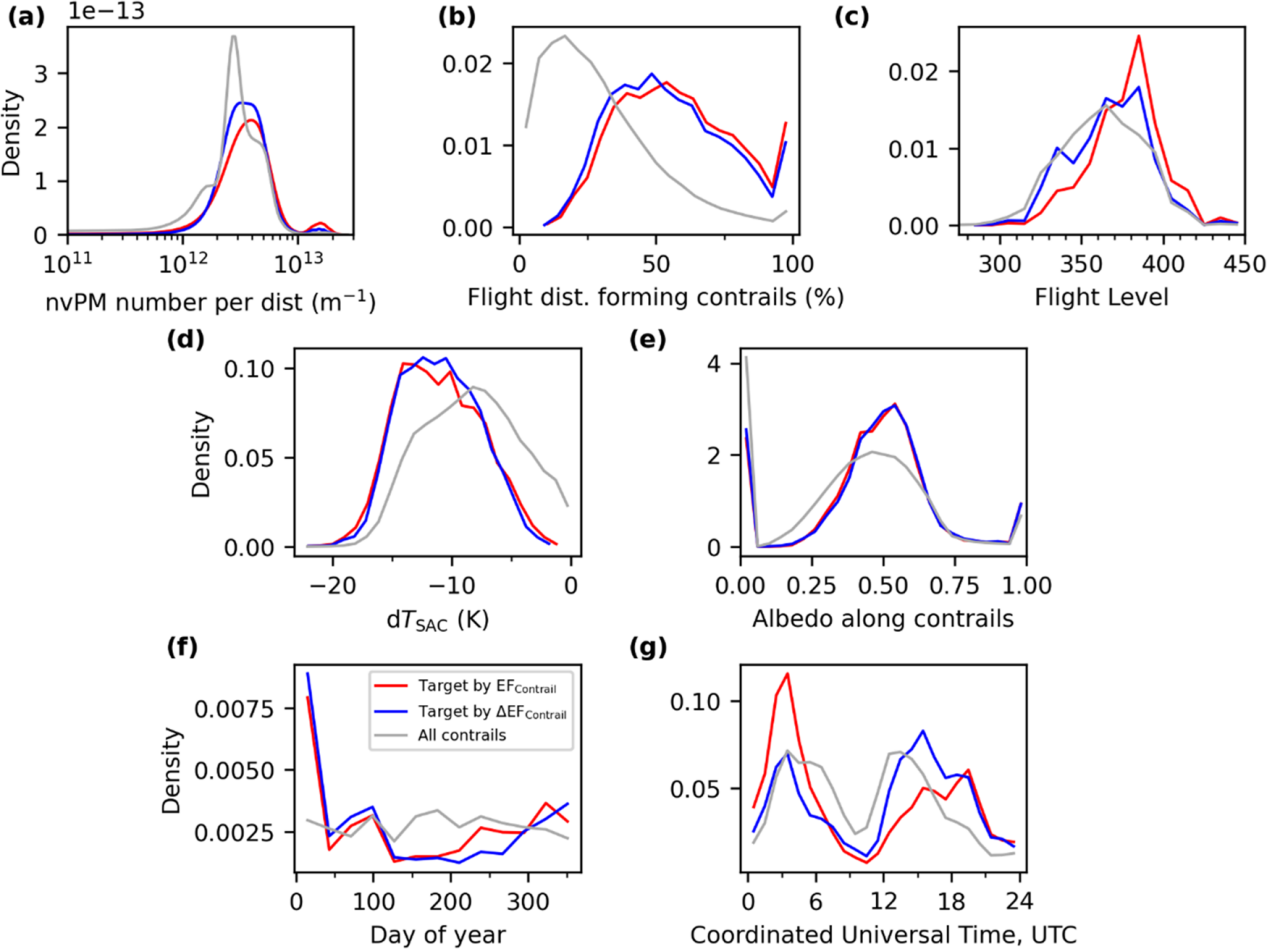
Probability density function of the trajectory, nvPM emissions, and meteorological conditions for all contrail-forming flights (gray lines), as well as the subset of flights that are targeted with SAF at a 50% blending ratio by descending order of their EF_contrail_ (red lines) or ΔEF_contrail_ (blue lines).

Figure 4 summarizes the characteristics of flights that are targeted with SAF with 50% *p*_blend_ using the two allocation strategies (i.e., targeting flights by order of EF_contrail_ or ΔEF_contrail_). SAF is generally recommended when the: (i) nvPM number emissions per flight distance, which varies by aircraft type,^41,47^ exceeds 2 × 10^12^ m^–1^; (ii) percentage of flight distance forming contrails exceeds 25%; (iii) cruising altitude is between 35,000 and 40,000 feet; (iv) difference between the ambient and SAC threshold temperature (d*T*_SAC_) is greater than 10 K; (v) albedo along the flight trajectory is above 0.4, indicating that contrails are formed above optically thick low-level water clouds; and/or (vi) during wintertime where the ISSR coverage is at its seasonal peak.^41^ Conditions (i), (iii), and (iv) can lead to strongly warming contrails because they reduce *r*_ice_ and increase the contrail lifetime,^41^ while condition (v) lowers the contrail SW RF′ because the incoming SDR would have been reflected by the low-level clouds even without the contrails.^41^ Contrails produced by low nvPM-emitting engines tend to have smaller EF_contrail_^32,41^ and are not selected for SAF deployment (Figure 4a). The key difference between the two allocation strategies is the time of day at which SAF is provided (Figure 4g). An allocation strategy by EF_contrail_ causes SAF to be predominantly deployed on eastbound flights (62% of flights with SAF), between 02:00 and 05:00 UTC, because the magnitude of EF_contrail_ during these times tends to be large relative to other time periods.^41^ However, this is suboptimal because the shorter contrail lifetime resulting from SAF could reduce the probability of contrails surviving until dawn where their cooling effect can partly offset their cumulative warming effects. In contrast, allocating SAF to flights with the highest ΔEF_contrail_ leads to an equal split in SAF distribution between eastbound (48%) and westbound flights (52%), and a higher proportion of SAF is deployed between 13:00 and 16:00 UTC because it can shorten the contrail lifetime such that the contrail persists only during daylight hours with a net cooling effect.

The reduction in annual CO_2_ EF (ranging between 0.12 and 0.96%, depending on the quantity of SAF and assumptions on the CO_2_ WTW emissions) does not vary between the different allocation strategies considered (Table S8). The relative contribution of the contrail cirrus component in reducing the EF_total_ (CO_2_ + contrails) is between 48 and 88% in the uniform distribution approach (SAF1) and increases to between 88 and 99% when targeted strategies with higher SAF blend ratios are used. Therefore, reductions in EF_total_ from the SAF allocation by ΔEF_contrail_ with a 50% *p*_blend_ (between −6.5 and −6.2%) is approximately 9 to 15 times larger than the baseline scenario (between −0.8 and −0.4%, SAF1) (Table S8), depending on the assumed reduction in CO_2_ life cycle emissions from SAF.

## 4 Implications

SAF supply is expected to be severely constrained in the coming decade while production facilities are ramped up.^11^ At present, only seven EU airports have a regular supply of SAF,^40^ and on an airline level, SAF is generally added into the existing fuel pipeline and uniformly distributed to a subset of flights with very low blending ratios.^61^ This study proposes that SAF be blended at higher ratios and deployed to a fraction of flights responsible for the most strongly warming contrails. We find that this can increase the overall climate benefits of SAF by a factor of 9–15 relative to a scenario in which SAF is uniformly distributed. Targeting flights using SAF with *p*_blend_ above 50% leads to smaller reductions in the EF_contrail_ relative to the scenario with 50% *p*_blend_ (Figure 3 and Supporting Information S3.1). Given the short-lived nature of contrail climate effects relative to CO_2_, an intelligent allocation of SAF offers the potential to rapidly reduce the overall climate impact of global aviation. Previous studies have shown that the annual EF_contrail_ is concentrated on a small percentage of flights^32,41^ and we expect these climate benefits to be valid when applied to other regions, but this should be a topic for future research.

We note that the contrail climate forcing is most sensitive to the corrections applied to the ERA5 HRES humidity fields,^41^ and simulations without humidity corrections approximately halved the contrail cirrus net RF in the baseline (from 235 to 121 mW m^–2^) and SAF100 (from 133 to 68.5 mW m^–2^) scenarios. While the relative difference between the contrail net RF in the baseline and SAF100 scenarios without humidity correction (235 vs 133 mW m^–2^ ,−43.4%) is consistent with the difference between these simulations with humidity correction (121 vs 68.5 mW m^–2^ ,−43.5%), the lower magnitude of EF_contrail_ means that the additional climate gains achieved from a targeted SAF strategy would be halved.

Future research priorities include: (i) a holistic quantification of meteorological, emissions, and contrail model uncertainties on the simulated contrail properties; (ii) comparisons between in situ contrail measurements and model estimates resulting from different fuel types to improve the model prediction quality; (iii) evaluating different distribution strategies in allocating the limited SAF supply (i.e., to specific airports, routes, and/or different segments on the flight) to maximize its climate benefits; and (iv) investigating the additional health and local air quality benefits that can be gained from the targeted SAF strategy.

## Supporting Information Available

nvPM EI*_n_* reductions due to SAF; fuel properties from different SAF blending ratios; fleetwide adoption of SAF (nvPM emissions; contrail properties; and comparison of results with existing studies), and targeted use of SAF (PDF)

## Accession Codes

Emissions and contrail model codes are available for scientific research upon request.

## Notes

Flight trajectory data is commercially sensitive and is available on reasonable request from NATS (responsible@nats.co.uk). This document used elements of Base of Aircraft Data (BADA) Family 4 Release 4.2 which has been made available by EUROCONTROL to Imperial College London. EUROCONTROL has all relevant rights to BADA. ©2019 The European Organisation for the Safety of Air Navigation (EUROCONTROL). EUROCONTROL shall not be liable for any direct, indirect, incidental, or consequential damages arising out of or in connection with this document, including the use of BADA. This document contains Copernicus Climate Change Service information 2022. Neither the European Commission nor ECMWF is responsible for any use of the Copernicus information.
