# Supplementary material for: Targeted Use of Sustainable Aviation Fuel to Maximize Climate Benefits
Source: Environ Sci Technol. 2022 Nov 17;56(23):17246–55. doi: 10.1021/acs.est.2c05781 (PMC9730838; doi:10.1021/acs.est.2c05781)
Supplement: Supplementary file 1 — es2c05781_si_001.pdf [file es2c05781_si_001.pdf]

Supporting Information

for

Targeted Use of Sustainable Aviation Fuel to Maximize Climate Benefits

Roger Teoh[1], Ulrich Schumann[2], Christiane Voigt[2,3], Tobias Schripp[4], Marc Shapiro[5], Zebediah Engberg[5], Jarlath Molloy[6], George Koudis[6] and Marc E.J. Stettler[1]*

[1] Department of Civil and Environmental Engineering, Imperial College London, London, SW7 2AZ, United Kingdom

[2] Institute of Atmospheric Physics, Deutsches Zentrum für Luft- und Raumfahrt, 82234 Oberpfaffenhofen, Germany

[3] Institute of Atmospheric Physics, University Mainz, 55099 Mainz, Germany

[4] Institute of Combustion Technology, Deutsches Zentrum für Luft- und Raumfahrt, 70569 Stuttgart, Germany

[5] Orca Sciences, 4110 Carillon Point, Kirkland, WA 98033, United States

[6] NATS, 4000 Parkway, Whiteley, Fareham, Hampshire, PO15 7FL, United Kingdom

* Corresponding author: m.stettler@imperial.ac.uk

Supporting Information Details:

- Number of pages: 22
- Number of figures: 11
- Number of tables: 8

Contents

S1 nvPM EI_n reductions due to sustainable aviation fuels 3

S2 Fuel properties from different SAF blending ratios 10

S3 Fleetwide adoption of SAF 12

S3.1 nvPM emissions 12

S3.2 Contrail properties 14

S3.3 Comparison with existing studies 16

S4 Targeted use of SAF 18

S1 nvPM EI_n reductions due to sustainable aviation fuels

Two different methodologies are available to estimate the change in nvPM EI_n from fuels with different hydrogen mass content (H_{fuel}). Brem et al.[1] used ground-based experimental measurements to develop a linear relationship between the percentage reduction in nvPM EI_n versus engine thrust settings ($\hat{F}$, in %) and H_{fuel},

$$\Delta \text{nvPM EI}_{\text{n}}[\%] = \left(\alpha_0 + \alpha_1 \hat{F}\right) \times \Delta H, \qquad \text{(S1)}$$

$$\text{where } \Delta H = H_{\text{SAF}} - H_{\text{ref}}.$$

$\alpha_0 = -114.21$ and $\alpha_1 = 1.06$ are fitted parameters[1], and ΔH is the difference in H_{fuel} between the SAF (H_{SAF}) and "reference" Jet A-1 fuel (H_{ref}). However, Brem et al.[1] highlighted that Eq. (S1) is only valid for $\hat{F} > 30\%$ and $\Delta H < 0.6$, and Figure S1 confirms that an extrapolation beyond these limits can lead to unrealistic values where $\Delta \text{nvPM EI}_{\text{n}} < -100\%$.

Alternatively, the standardised fuel composition correction model was also developed for the ICAO Committee on Aviation Environmental Protection (CAEP/11) to account for the variability/differences in the properties of conventional kerosene fuel, so that the measured nvPM EI_n corresponds to a H_{fuel} of 13.8% (Appendix 6.2.2 of the ICAO Annex 16 Vol. II)[2],

$$\text{Corrected nvPM EI}_{\text{n}} = \text{nvPM EI}_{\text{n}} \times k_{\text{fuel\_N}}, \qquad \text{(S2)}$$

$$\text{where } k_{\text{fuel,N}} = \exp\left\{\left(0.99 \frac{F}{F_{00}} - 1.05\right)(13.8 - H_{\text{fuel}})\right\}.$$

$k_{\text{fuel,N}}$ is the fuel composition correction factor for the nvPM EI_n and $\frac{F}{F_{00}}$ is the engine thrust settings (in decimals). Figure S2 shows the estimated $\Delta \text{nvPM EI}_{\text{n}}$ from the ICAO CAEP/11 model across a range of $\hat{F}$ and ΔH relative to a H_{fuel} of 13.8%. We note that Eq. (S2) is only valid for an allowable H_{fuel} range of between 13.4 and 14.3% (Appendix 4 of the ICAO Annex

16 Vol. II)[2], and using it to estimate the change in ΔnvPM EI_n from SAF with high blend ratios (H_{fuel} > 14.3%) could therefore lead to inaccuracies.

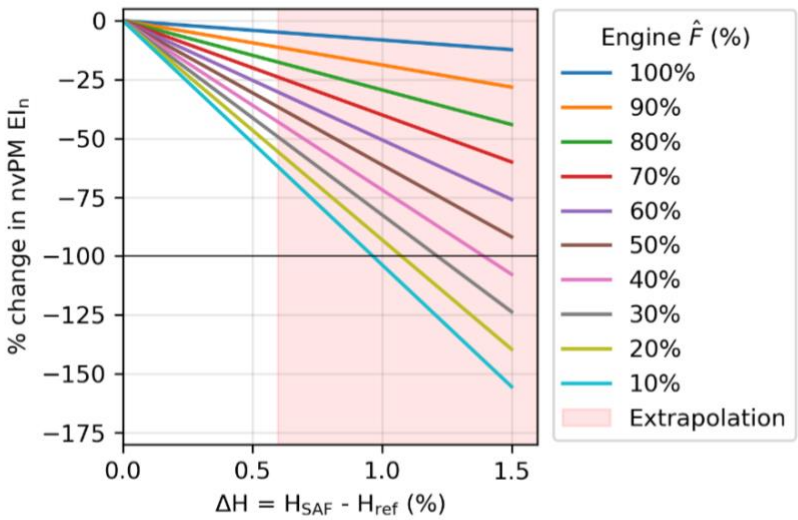

Figure S1: The change in nvPM EI_n (%) as a function of the ΔH and $\hat{F}$ that was developed by Brem et al.[1], as outlined in Eq. (S1). The shaded region (in red) represents estimates are extrapolations from the available measurements.

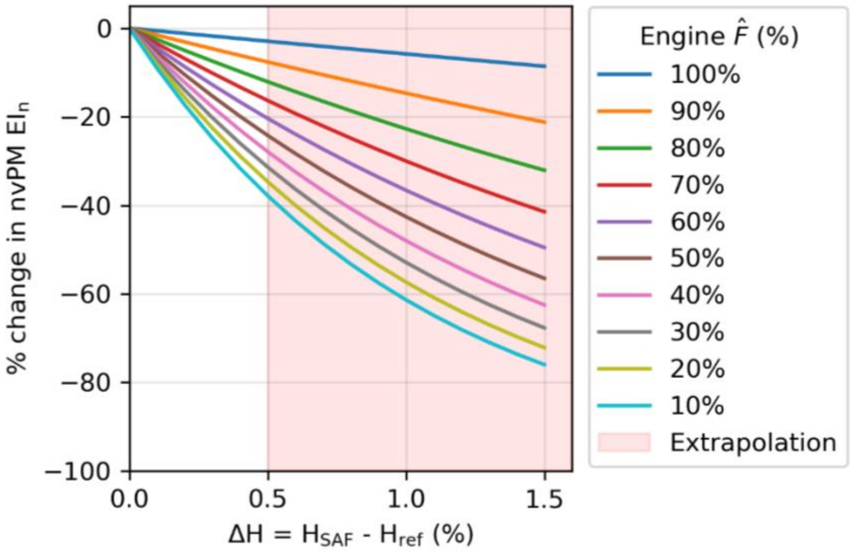

Figure S2: The change in nvPM EI_n (%) as a function of ΔH and $\hat{F}$ that was developed for the ICAO CAEP/11, as outlined in Eq. (S2). The shaded region (in red) represents estimates are extrapolations from the available measurements.

In this study, we extend the methodology of Brem et al.[1] to account for its known limitations. The extended methodology, outlined in Eq. (1) in the main text and visualised in Figure S3, utilises latest measurements from the NASA ACCESS[3] and ECLIF II/ND-MAX[4–6] experimental campaigns, which measured the nvPM EI_n emitted by SAF with higher H_{fuel} of

up to 14.7% corresponding to a ΔH of up to 1.1%. Therefore, this enables our extended fuel composition correction model to be applied to estimate the nvPM EI_n from SAF with higher H_{fuel} (and blending ratios).

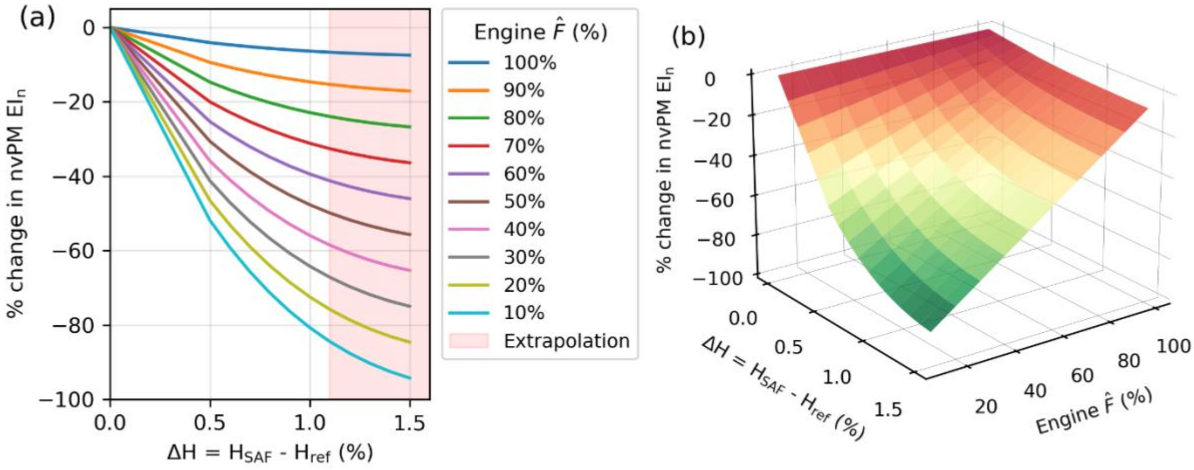

Figure S3: Visualisation of Eq. (1) in the main text, which extends the methodology of Brem et al.[1] to estimate the change in nvPM EI_n (%) for a wider range of ΔH. The shaded region (red) in (a) represents estimates are extrapolations from the available measurements.

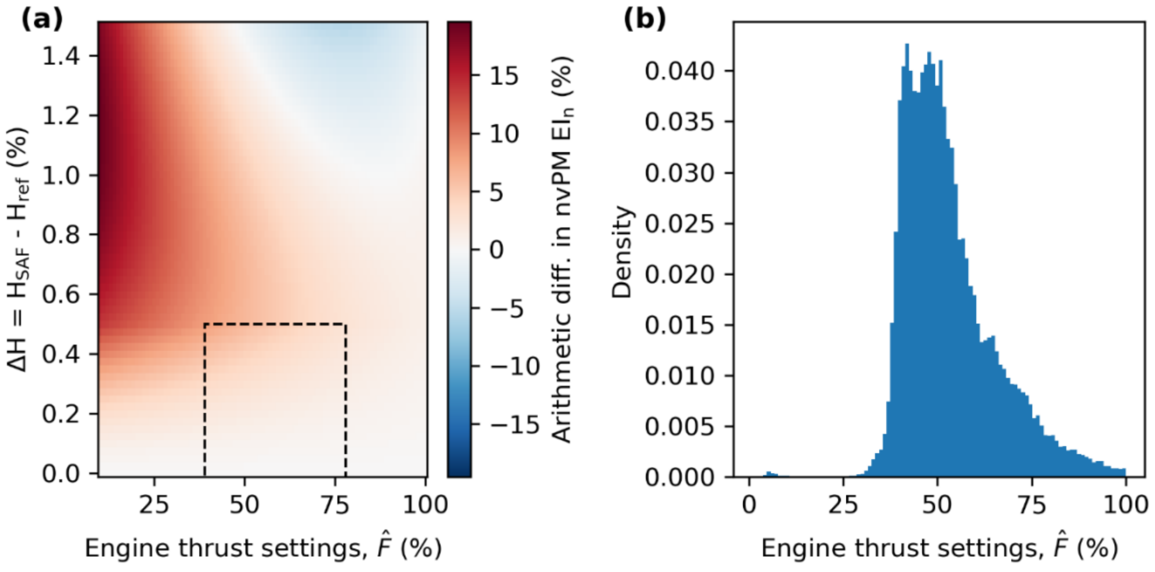

Figure S4: (a) Arithmetic difference in $\Delta nvPM\ EI_n$ between the extended fuel correction model (Eq. 1 in the main text) and the standardised fuel composition correction model for the ICAO CAEP/11 standard (Eq. S2), where the bounding box represents the ΔH for which the ICAO CAEP/11 method is valid, i.e., between -0.4% and +0.5%, and to the range of $\hat{F}$ that is typically used at cruise conditions as shown in the histogram in (b). For (b), $\hat{F}$ is calculated as $\frac{\dot{m}_f^{MSL}}{\dot{m}_{f,max}^{MSL}}$, where the fuel mass flow rate at mean sea level conditions ($\dot{m}_f^{MSL}$) is estimated using Eq. 2 in the main text, while fuel mass flow rate at maximum engine thrust settings ($\dot{m}_{f,max}^{MSL}$) for different engines is provided by the ICAO EDB[22].

We compare the differences in the estimated $\Delta\mathrm{nvPM\,EI_n}$ between the extended fuel composition correction model (Eq. 1 in the main text) and the ICAO CAEP/11 (Eq. S2). Figure S4a shows that arithmetic differences between the two models range between -5% and +20% across the full range of $\hat{F}$ and ΔH. However, the arithmetic difference between the two models reduces to between 0% and +8% when we constrain ΔH for which the ICAO CAEP/11 method is valid, i.e., ΔH of between -0.4% and +0.5%, and to the range of $\hat{F}$ that is typically used in cruise conditions, i.e., between 39% (5th percentile) and 78% (95th percentile) (Figure S4b).

The estimated $\Delta\mathrm{nvPM\,EI_n}$ from both models are also compared against: (i) ground measurements from the A-PRIDE[1], EMPAIREX[7], and ECLIF2/ND-MAX[4] campaigns; and (ii) cruise measurements from the NASA ACCESS and ECLIF2/ND-MAX campaigns. Tables S1 to S5 compiles the fuel properties, engine operating conditions, as well as the measured and estimated nvPM $\mathrm{EI_n}$ from the five experimental campaigns. We note that the ECLIF/ND-MAX[6] campaign at cruise measured the nvPM $\mathrm{EI_n}$ for different fuel types independently without fixing the fuel mass flow rate, and therefore, we used data from the International Civil Aviation Organization (ICAO) Aircraft Emissions Databank (EDB)[8] and the methodology of Teoh et al.[9] to estimate the nvPM $\mathrm{EI_n}$ that would have been emitted under the "reference" Jet A-1 fuel, and then scale the nvPM $\mathrm{EI_n}$ from SAF using the two independent methodologies. The coefficient of determination (R^2) and normalised mean bias (NMB) from both models are presented in Figures S5 and S6. It shows that: (i) the performance of both models is comparable when compared against ground measurements (R^2 = 0.84 and NMB = +28% for our extended model, vs. R^2 = 0.78 and NMB = +6.8% for the ICAO CAEP/11); but (ii) our extended fuel composition model outperforms the ICAO CAEP/11 approach when compared against cruise measurements (R^2 = 0.83 and NMB = -3.2% for the extended model, vs. R^2 = 0.03 and NMB = -13% for the ICAO CAEP/11). For the comparison with cruise measurements, we note that the negative bias in the estimated $\Delta\mathrm{nvPM\,EI_n}$ from the ICAO CAEP/11 (NMB = -13%) is

consistent with the results shown in Figure S4a, where the estimated ΔnvPM EI_n from the ICAO CAEP/11 is between 0% and 8% smaller than those estimated from our extended fuel composition correction model. This comparison provides supporting evidence that our extended fuel composition correction model (Eq. 1 in the main text) is applicable for both ground and cruise conditions.

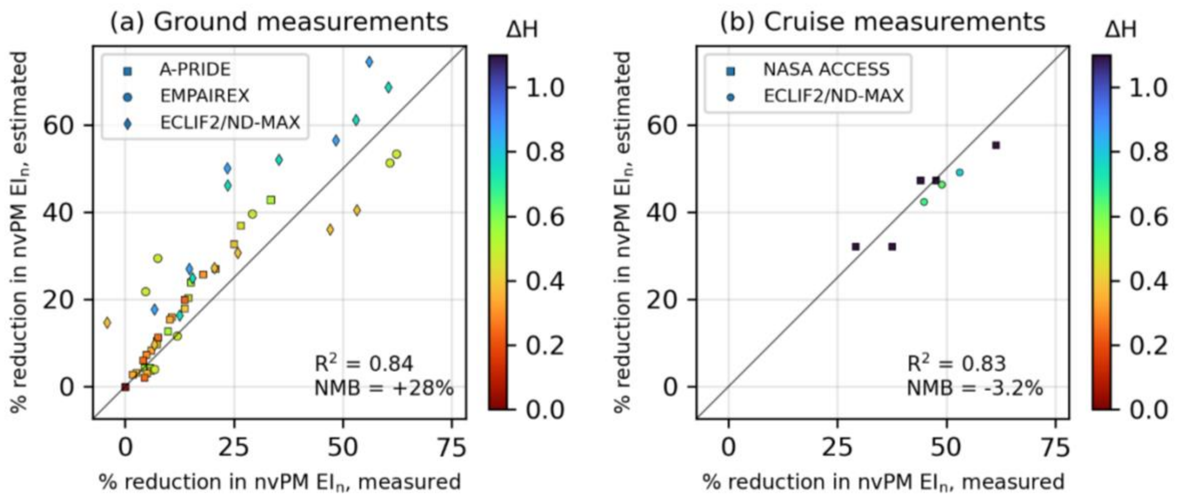

Figure S5: Evaluation of the extended fuel composition correction model (Eq. 1 in the main text) that is used to estimate the ΔnvPM EI_n that arise from the use of SAF relative to: (a) ground; and (b) cruise measurements from four different experimental campaigns. Detailed data tables are shown in Tables S1 to S5.

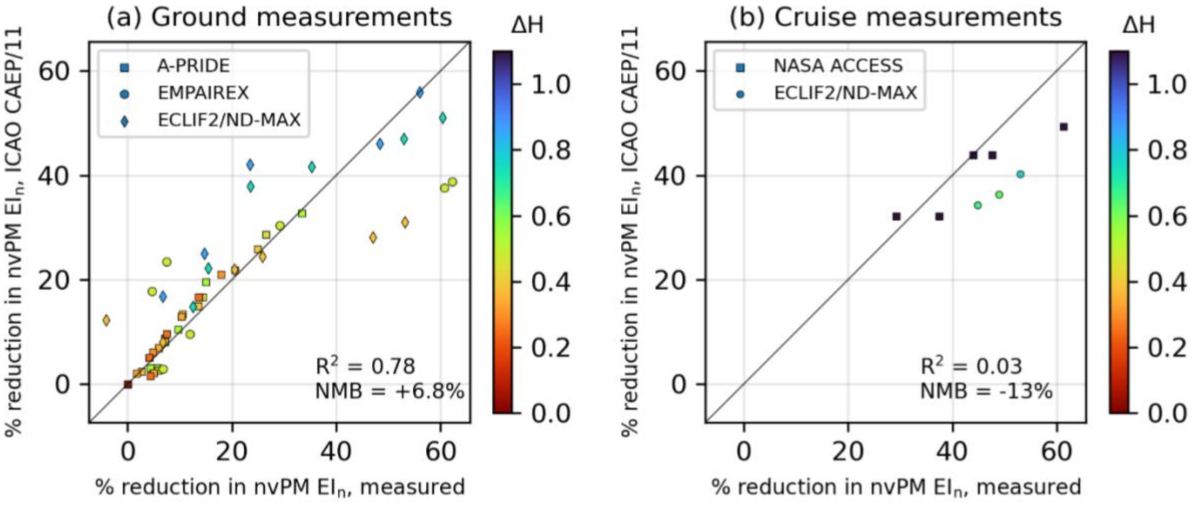

Figure S6: Evaluation of the standardised fuel composition correction model from the ICAO CAEP/11 (Eq. S2) that is used to estimate the ΔnvPM EI_n that arise from the use of SAF relative to: (a) ground; and (b) cruise measurements from four different experimental campaigns.

Table S1: Comparison of the percentage change in nvPM EI_n that result from burning fuels with different fuel hydrogen mass content (H_{fuel}). The dataset consist of ground measurements from the A-PRIDE experimental campaign, and is extracted from Figure 4d of Brem et al.[1].

$\hat{F}$ (%)	H_{fuel} (%)	nvPM EI_n, H_{fuel} (x 10^{14} kg^{-1})	nvPM EI_n, H = 14.31% (x 10^{14} kg^{-1})	ΔH (%)[a]	ΔnvPM EI_n, measured (%)	ΔnvPM EI_n, est. (%)[b]
30	13.78	1.65	1.10	0.53	-33.39	-42.84
30	13.86	1.50	1.10	0.45	-26.53	-36.92
30	13.91	1.47	1.10	0.40	-24.93	-32.72
30	13.98	1.39	1.10	0.33	-20.64	-27.05
30	14.00	1.34	1.10	0.31	-17.92	-25.77
30	14.07	1.27	1.10	0.24	-13.62	-19.91
30	14.31	1.10	1.10	0.00	0.00	0.00
65	13.77	4.59	3.90	0.54	-14.97	-23.85
65	13.86	4.56	3.90	0.45	-14.43	-20.33
65	13.91	4.51	3.90	0.40	-13.59	-18.02
65	13.96	4.36	3.90	0.35	-10.57	-15.99
65	13.97	4.35	3.90	0.34	-10.27	-15.46
65	14.06	4.22	3.90	0.25	-7.56	-11.29
65	14.31	3.90	3.90	0.00	0.00	0.00
85	13.78	3.88	3.50	0.53	-9.72	-12.66
85	13.87	3.77	3.50	0.44	-7.19	-10.59
85	13.90	3.77	3.50	0.41	-7.20	-9.77
85	13.97	3.72	3.50	0.34	-6.00	-8.30
85	14.01	3.68	3.50	0.30	-4.87	-7.31
85	14.06	3.65	3.50	0.25	-4.08	-5.99
85	14.31	3.50	3.50	0.00	0.00	0.00
100	13.77	3.14	3.00	0.54	-4.36	-4.32
100	13.77	3.18	3.00	0.54	-5.73	-4.32
100	13.87	3.19	3.00	0.44	-5.87	-3.61
100	13.91	3.08	3.00	0.40	-2.63	-3.25
100	13.94	3.16	3.00	0.37	-4.97	-3.03
100	13.97	3.05	3.00	0.34	-1.67	-2.79
100	14.06	3.14	3.00	0.25	-4.42	-2.05
100	14.31	3.00	3.00	0.00	0.00	0.00

[a]: $\Delta H = 14.31 - H_{fuel}$

[b]: Calculated using Eq. (1) in the main text.

Table S2: Comparison of the change in nvPM EI_n that result from burning conventional kerosene versus SAF. The dataset consist of ground measurements from the EMPAIREX campaign, and is downloaded from the Supporting Information of Durdina et al.[7] and aggregated by engine thrust settings ($\hat{F}$).

EMPAIREX 1, Ground[7]	Measured nvPM EI_n (x 10^{14} kg^{-1})		ΔnvPM EI_n, measured (%)	ΔnvPM EI_n, est. (%)[a]
$\hat{F}$ (%)	Jet A-1 (H = 13.57%)	68% Jet A-1 + 32% HEFA-SPK (H = 14.05%)		
3	20.5 ± 2.30	7.72 ± 2.00	-62.3	-53.3
7	2.98 ± 0.295	1.17 ± 0.080	-60.8	-51.3
30	4.40 ± 0.270	3.11 ± 0.459	-29.2	-39.6
50	6.03 ± 1.38	5.58 ± 0.317	-7.51	-29.4
65	14.8 ± 2.06	14.1 ± 0.700	-4.72	-21.7
85	15.1 ± 0.691	13.2 ± 0.222	-12.0	-11.6
100	12.3 ± 0.110	11.5 ± 0.404	-6.83	-3.94

[a]: Calculated using Eq. (1) in the main text. $\Delta H = 14.05 - 13.57 = 0.48$.

Table S3: Comparison of the percentage change in nvPM EI_n that result from burning conventional kerosene versus SAF. The dataset consist of ground measurements from the ECLIF2/ND-MAX campaign, and is provided by Schripp et al.[4] and aggregated by engine thrust settings ($\hat{F}$).

ECLIF2/ND-MAX, Ground		Measured nvPM EI_n (x 10^{14} kg^{-1})			
**Fuel mass flow rate (kg s^{-1})**	**$\hat{F}$ (%)**	**Ref3 (H = 13.65%)**	**SAJF1 (H = 14.4%)**	**SAJF2 (H = 14.51%)**	**SAJF3 (H = 14.04%)**
0.11 ± 0.001	10	6.37 ± 0.83	2.52 ± 0.31	2.80 ± 2.25	2.98 ± 1.01
0.22 ± 0.003	20	5.82 ± 0.73	2.74 ± 0.09	-	3.08 ± 0.20
0.35 ± 0.01	34	5.78 ± 0.34	3.74 ± 0.07	2.98 ± 0.49	4.28 ± 0.25
0.44 ± 0.007	42	5.56 ± 0.21	4.25 ± 0.07	4.25 ± 0.23	4.42 ± 0.51
0.76 ± 0.015	72	3.80 ± 0.20	3.21 ± 0.07	3.24 ± 0.12	3.95 ± 0.12
0.89 ± 0.02	85	2.85 ± 0.07	2.50 ± 0.11	2.66 ± 0.08	2.66 ± 0.05

ECLIF2/ND-MAX, Ground	ΔnvPM EI_n (%)					
	**Ref3 vs. SAJF1 (ΔH = 0.75%)**		**Ref3 vs. SAJF2 (ΔH = 0.86%)**		**Ref3 vs. SAJF3 (ΔH = 0.39%)**	
**$\hat{F}$ (%)**	**Measured**	**Estimated[a]**	**Measured**	**Estimated[a]**	**Measured**	**Estimated[a]**
10	-60.44	-68.58	-56.04	-74.44	-53.22	-40.41
20	-53.01	-61.07	-	-	-47.08	-35.99
34	-35.32	-51.98	-48.40	-56.41	-25.89	-30.63
42	-23.58	-46.09	-23.49	-50.02	-20.52	-27.16
72	-15.50	-24.84	-14.76	-26.96	4.08	-14.64
85	-12.56	-16.28	-6.78	-17.67	-6.78	-9.59

[a]: Calculated using Eq. (1) in the main text.

Table S4: Comparison of the estimated nvPM EI_n from SAF using cruise measurement data from the NASA Alternative Fuel Effects on Contrails and Cruise Emissions Study (ACCESS) experimental campaign[3].

NASA ACCESS, Cruise[3]				Measured nvPM EI_n (x 10^{14} kg^{-1})		ΔnvPM EI_n, measured (%)	ΔnvPM EI_n, est. (%)[b]
**Engine No. (Thrust)**	**$\dot{m}_f^{Cruise}$ (kg s^{-1})**	**$\dot{m}_f^{MSL}$ (kg s^{-1})[a]**	**$\hat{F}$ (%)**	**Medium sulphur content Jet A fuel (H = 13.6%)**	**50:50 HEFA:low sulphur content Jet A fuel blend (H = 14.7%)**		
2 (High)	0.373	0.599	70.5	7.64 ± 0.15	5.41 ± 0.15	-29.19	-32.17
2 (Medium)	0.280	0.449	7	5.00 ± 0.14	2.62 ± 0.07	-47.60	-47.36
2 (Low)	0.231	0.371	30	4.50 ± 0.23	4.15 ± 0.63	-7.78[c]	-55.36
3 (High)	0.373	0.599	50	6.30 ± 0.13	3.94 ± 0.12	-37.46	-32.17
3 (Medium)	0.280	0.449	65	3.18 ± 0.14	1.78 ± 0.06	-44.03	-47.36
3 (Low)	0.231	0.371	100	2.82 ± 0.08	1.09 ± 0.08	-61.35	-55.36

[a]: Calculated using Eq. (2) in the main text. Air temperature estimated from the pressure altitude by assuming the ICAO standard atmosphere. Mach number set to be 0.6.

[b]: Calculated using Eq. (1) in the main text.

[c]: Data point is identified as an outlier and not included in the comparison.

Table S5: Comparison of the estimated nvPM EI_n from SAF using cruise measurement data from the Emission and Climate Impact of Alternative Fuel (ECLIF/ND-MAX) campaign[6].

ECLIF2/ND-MAX, Cruise[6]	Ref 2	SSF1	SAF1	SAF2
Altitude (m)	10670	10364	9726	9656
Pressure altitude (m)	23835	24996	27564	27858
Air temperature (K)	215	220	218	216
Mach number	0.65	0.58	0.76	0.76
Hydrogen mass content (%)	13.73	14.36	14.4	14.51
Measured nvPM EI_n (x 10^{15} kg^{-1})	4.9 ± 0.6	2.5 ± 0.2	2.7 ± 0.6	2.3 ± 0.6
Estimated nvPM EI_n, Jet A-1 (x 10^{15} kg^{-1})[a]	5.517	6.693	6.183	6.187
ΔnvPM EI_n, measured (%)	-	-48.98	-44.90	-53.06
Fuel mass flow rate (kg s^{-1})	0.3278	0.2278	0.3144	0.3031
Equivalent fuel mass flow rate at MSL (kg s^{-1})[b]	0.4983	0.3542	0.4494	0.4138
Engine thrust settings ($\hat{\boldsymbol{F}}$)	0.475	0.3376	0.4284	0.3945
ΔH (%)	-	0.63	0.67	0.78
ΔnvPM EI_n, estimated (%)[c]	-	-46.30	-42.34%	-49.09%

[a]: Estimated using the nvPM EI_n measurements from the ICAO EDB, where it is interpolated relative to the non-dimensional engine thrust settings[9].

[b]: Calculated using Eq. (2) in the main text.

[c]: Calculated using Eq. (1) in the main text.

S2 Fuel properties from different SAF blending ratios

The fuel properties of conventional "reference" fuels and SAF with different blending ratios are compiled from the literature and presented in Table S6. We use the compiled dataset to develop a linear relationship between the SAF blending ratio (p_{blend}, in %) versus the fuel hydrogen mass content (H_{fuel}, in %) and lower calorific value (LCV in J kg^{-1}) (Figure S7). On this basis,

the following equations are used to approximate the SAF fuel hydrogen content (H_{SAF}) and LCV (LCV_{SAF}) for different p_{blend},

$$H_{\text{SAF}}[\%] = H_{\text{ref}} + 0.015 \times p_{\text{blend}}, \quad \text{(S3)}$$

$$\text{LCV}_{\text{SAF}} = \text{LCV}_{\text{ref}} + 10700 \times p_{\text{blend}}, \quad \text{(S4)}$$

where we assume H_{ref} = 13.8% and LCV_{ref} = 43.1 ×10^6 J kg^{-1} respectively for the "reference" Jet A-1 fuel[7,10]; and $H_{\text{SAF=100\%}}$ = 15.3% and $\text{LCV}_{\text{SAF=100\%}}$ = 44.2 ×10^6 J kg^{-1} respectively for a

fully synthetic SAF (p_{blend} = 100%)[11,12]. The $\mathrm{EI_{H_2O}}$ is also assumed to increase proportionally[11] with H_{SAF},

$$\mathrm{EI_{H_2O,SAF}} = \mathrm{EI_{H_2O,ref}} \times \left(\frac{H_{\mathrm{SAF}}}{H_{\mathrm{ref}}}\right), \tag{S5}$$

where $\mathrm{EI_{H_2O,ref}} = 1.237$ kg kg^{-1} (ref.[11]). Table 1 in the main text shows the H_{SAF}, $\mathrm{LCV_{SAF}}$ and $\mathrm{EI_{H_2O,SAF}}$ that is assumed for the six simulation runs with different SAF p_{blend} (1%, 10%, 30%, 50%, 70% and 100% blending ratios).

Table S6: Compilation of the fuel properties for traditional Jet A-1 fuels and SAF with different blending ratios from various experimental campaigns.

(a) Conventional "reference" fuels	Experimental Campaign	Lower Calorific Value, LCV (MJ kg^{-1})	Hydrogen mass content (%)	Ref.
JP-8	AAFEX-II	-	13.5	[1]
Low-sulfur-content Jet A	ACCESS	43.15 ± 0.06	13.8 ± 0.2	[2]
Jet A-1 (Ref1)	ECLIF	42.8	14.1	[3]
Jet A-1 (Ref1)	ECLIF/ND-MAX	42.80 ± 0.02	13.67 ± 0.14	[4]
Jet A-1 (Ref2)	ECLIF	43.2	14.1	[3]
Jet A-1 (Ref3)	ECLIF/ND-MAX	43.14 ± 0.01	13.65 ± 0.05	[4]
Jet A-1 (Ref4)	ECLIF/ND-MAX	43.34 ± 0.01	14.08 ± 0.18	[4]
Jet A-1	EMPAIREX 1	43.3	13.68	[5]

(b) HEFA-SPK Fuel Blends	Experimental Campaign	Lower Calorific Value, LCV (MJ kg^{-1})	Hydrogen mass content (%)	Ref.
50% JP-8 + 50% HEFA-SPK	AAFEX-II	-	14.4	[1]
50% Low-sulfur Jet A + 50% HEFA-SPK	ACCESS	43.52 ± 0.04	14.7 ± 0.2	[2]
51% Ref3 + 49% HEFA-SPK	ECLIF/ND-MAX	43.63 ± 0.01	14.40 ± 0.07	[4]
68% Jet A-1 + 32% HEFA-SPK	EMPAIREX 1	43.6	14.05	[5]
70% Ref4 + 30% HEFA-SPK	ECLIF/ND-MAX	43.63 ± 0.01	14.51 ± 0.04	[4]
100% HEFA-SPK	AAFEX-II	-	15.3	[1]
100% HEFA-SPK	-	44.2	-	[6]

(c) FT-SPK Fuel Blends	Experimental Campaign	Lower Calorific Value, LCV (MJ kg^{-1})	Hydrogen mass content (%)	Ref.
55% Ref2 + 45% FT-SPK	ECLIF	43.54	14.7	[3]
59% Ref1 + 41% FT-SPK	ECLIF	43.496	-	[3]
59% Ref2 + 41% FT-SPK	ECLIF/ND-MAX	43.50 ± 0.02	14.36 ± 0.02	[4]
86% Ref1 + 14% FT-SPK	ECLIF	43.301	14.2	[3]
100% FT-SPK	AAFEX-II	-	15	[1]

[1] Moore et al.[12]; [2] Moore et al.[3]; [3] Schripp et al.[13]; [4] Voigt et al.[6]; [5] Durdina et al.[7]; [6] Gierens et al.[11]

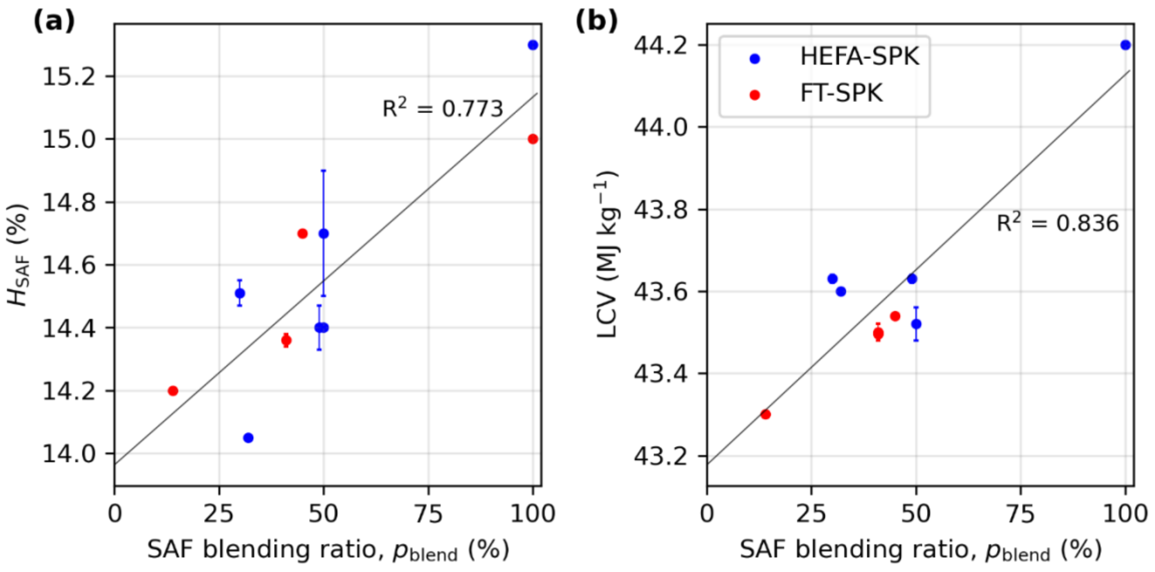

Figure S7: Linear relationship between the SAF blending ratio versus the: (a) fuel hydrogen mass content (H_{SAF}); and (b) fuel lower calorific value (LCV). Error bars denote precision errors from experimental measurements with one standard deviation. Detailed data tables are in Table S6.

We note the small deviations in the different fuel properties around the assumed linear trendline, for example, the H_{fuel} measured from SAF's with the same blending ratio can differ by up to ~0.5% (Figure S7a). This phenomenon likely arises from: (i) variations in the H_{fuel} of different conventional "reference" fuels (13.5 – 14.1%, Table S6) that was used to blend with the SAF; and (ii) the different technology pathways that was used to produce the SAF (i.e., HEFA-SPK and FT-SPK). Therefore, the stated H_{SAF} values for a given p_{blend} in Table 1 in the main text is only valid for this study, and variations in the H_{SAF} for a given p_{blend} are to be expected in different measurement campaigns and modelling studies depending on the composition of the reference fuel and SAF.

S3 Fleetwide adoption of SAF

S3.1 nvPM emissions

For aircraft types with nvPM measurements provided by the ICAO EDB, the nvPM EI_n for each waypoint is estimated according to Teoh et al.[9], which linearly interpolates the nvPM EI_n relative to the ratio of turbine inlet to compressor inlet temperatures (T_4/T_2). T_4/T_2 is a non-

dimensional measure of engine thrust settings that captures the differences in engine operations at ground and cruise conditions[10]. We note that the nvPM EI_n for certain engine combustor types such as double annular combustors (DAC) and twin annular premixing swirler (TAPS) experience a step change in their emissions profile, where the nvPM EI_n emitted in high $\hat{F}$ (lean-burn phase, which is activated above a certain $\hat{F}$) can be three to four orders of magnitude lower than those emitted at low $\hat{F}$ (rich-burn phase, ~10^{15} kg^{-1})[14,15].

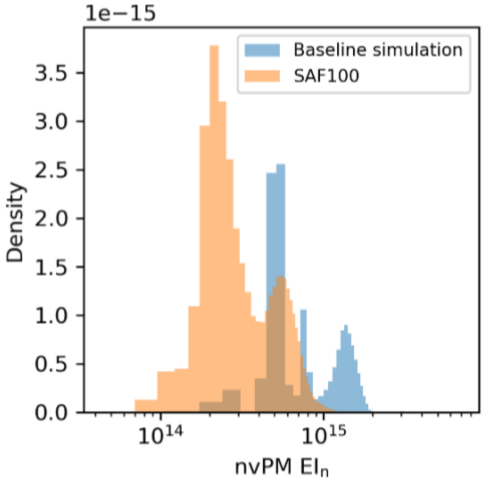

Figure S8: Probability density function of the mean nvPM EI_n for each flight in the baseline simulation and the simulation with fully synthetic SAF (SAF100).

In this study, we do not incorporate this step change in nvPM emissions for DAC and TAPS engines and instead, linearly interpolate the nvPM EI_n from the four data points provided by the ICAO EDB because the transition point between the "rich-burn" and "lean-burn" phase is not publicly available. However, we do not expect this assumption to change our simulation results because the: (i) nvPM EI_n inputs to the contrail cirrus prediction model (CoCiP)[16] is constrained to a lower bound of 10^{13} kg^{-1} to account for uncertainties and the potential activation of ambient aerosols and organic volatile particles into contrail ice crystals (refer to Figure 3 of Kärcher[17]); and (ii) only ~0.5% of all flights have a mean nvPM EI_n that is below 10^{14} kg^{-1} in the simulation with fully synthetic SAF (SAF100), as shown in Figure S8. The small number of flights with nvPM $EI_n < 10^{14}$ kg^{-1} (~0.5%) can be attributed to the low usage of aircraft types that are powered by the TAPS combustor (Boeing 737-MAX, 747-800, 787-

10 and the Airbus A320neo) over the North Atlantic (refer to Figures S6 and S7 of Teoh et al.[9]). Therefore, for all SAF simulations, the mean nvPM EI_n are in the "soot-rich" regime[17] and exceeds 10^{13} kg^{-1} by more than one order of magnitude (Table 1 in the main text).

S3.2 Contrail properties

The probability density functions in Figure S9 show the change in persistent contrail formation and contrail energy forcing ($EF_{contrail}$) for all contrail-forming flights when SAF with different blending ratios are used. Figure S10 provides a visualisation of the ice supersaturated regions (ISSR) that is encountered by two flights and the locations where contrails are formed for different fuel types, where the additional contrails formed by SAF are generally found at the edges of ISSR where ice supersaturation is weak. Figure S11 provides an example of the change in contrail optical depth ($\tau_{contrail}$) and coverage area between the baseline simulation (conventional fuel) versus the simulation where fully synthetic SAF is adopted by the fleet.

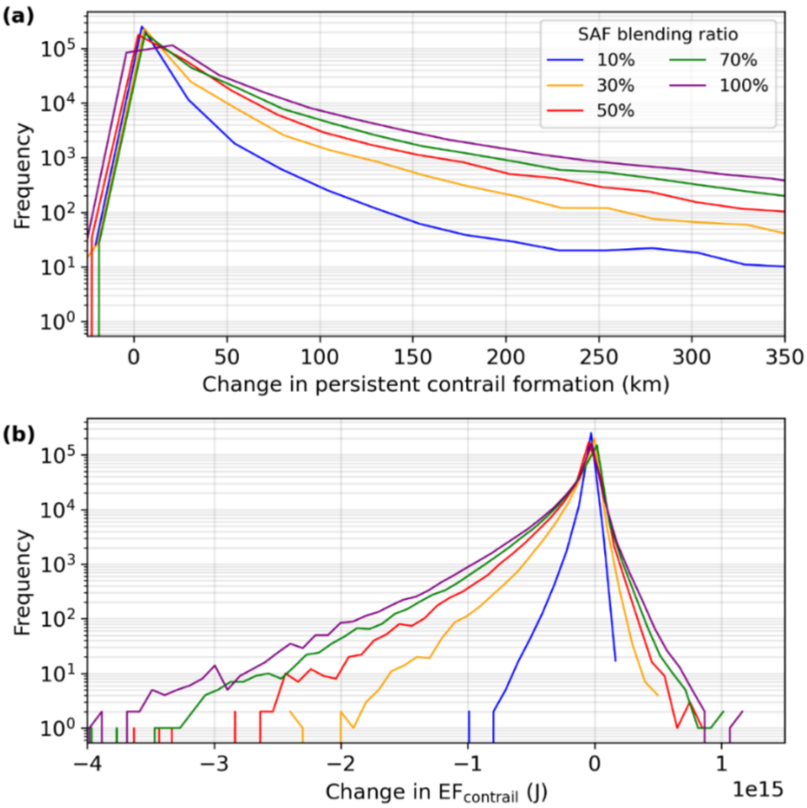

Figure S9: Probability density function of the change in (a) persistent contrail formation; and (b) total $EF_{contrail}$ for all contrail-forming flights (*n* = 267,076) when SAF with different blending ratios are used. The changes in contrail properties are relative to the baseline simulation with conventional fuels.

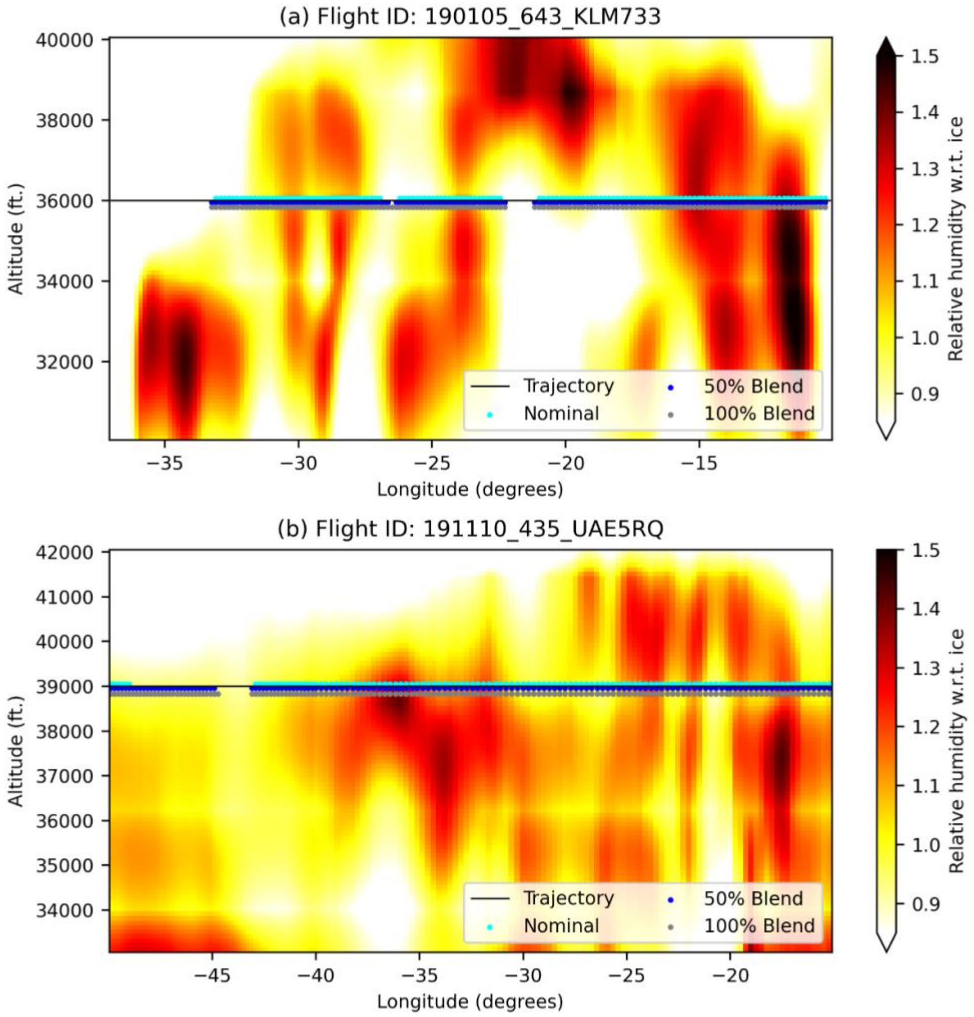

Figure S10: The flight trajectory (black lines), location of ISSRs (colour bars), and the resulting contrail locations when the two example flights are provided with conventional kerosene fuels (cyan), SAF with a 50% (blue) and 100% blending ratio (grey).

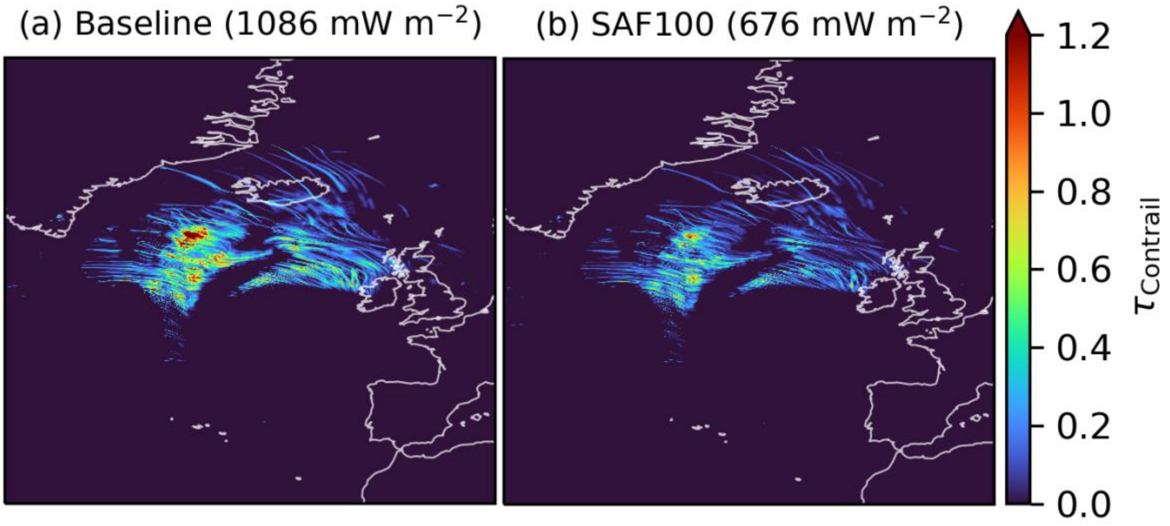

Figure S11: Gridded contrail cirrus optical depth ($\tau_{contrail}$) over the North Atlantic under clear sky conditions at 18-Sept-2019 14:00:00 (UTC), where the fleet is powered by: (a) conventional kerosene fuel (baseline simulation); and (b) fully synthetic SAF (SAF100). Basemap plotted using Cartopy 0.20.2 (C) Natural Earth; license: public domain.

S3.3 Comparison with existing studies

We compare the difference in contrail properties between the baseline scenario and SAF100 (Table 2 in the main text) relative to results from: (i) Caiazzo et al.[18], who simulated the impacts of SAF on contrails over the United States in 2006; (ii) Burkhardt et al.[19], who simulated contrails globally with differences in soot number emissions;(iii) Bock & Burkhardt[20], who simulated the global contrail effects resulting from SAF and improving engine efficiency for air traffic and meteorological conditions in 2050; and (iv) Schumann et al.[21], who simulated contrails globally and evaluated the sensitivity of various contrail properties to the inputs of nvPM EI_n. Table S7 summarises the percentage differences in simulated contrail properties between the different studies.

Table S7: Comparison of the change in the simulated contrail properties from this study relative to existing studies. The percentage change in contrail properties from all the studies reported below arise from the use of SAF and/or assumption of a lower nvPM EI_n to approximate the effects of SAF.

	**This study**	**Caiazzo et al.[18]**	**Burkhardt et al.[19]**	**Bock & Burkhardt[20]**	**Schumann et al.[21]**
**Study domain**	North Atlantic	USA	Global	Global (2050)	Global
**nvPM EI_n**	-52%	-75%	N/A	N/A	-50%
**Dist. forming persistent contrails**	+5%	+8%	N/A	N/A	N/A
**Contrail ice particle number**	-55%	-75%	-80%	-50%	N/A
**Contrail ice crystal size**	+26%	+58%	N/A	N/A	N/A
**$\tau_{contrail}$**	-22%	-29%	-49%	-30%	-21%
**Contrail cirrus coverage**	-41%	N/A	-41%	-15%	-23%
**Contrail net RF**	-44%	(-4, +18)%*	-50%	-14%	-39%

* The reported values from Caiazzo et al.[18] likely represent the mean contrail net RF', instead of the annual mean contrail cirrus net RF.

All four studies assumed a constant nvPM EI_n for all waypoints, while we account for variations in nvPM EI_n from different aircraft types (Section 2.2), and the mean reduction in nvPM EI_n from SAF (~52%) is a function of $\hat{F}$ and H_{SAF} (Section 2.3). We estimate a slightly smaller increase in flight distance forming persistent contrails (+5.0%) when compared with

Caiazzo et al.[18] (+8.0%), and this difference could be due to differences in the study domain and meteorological inputs (NOAA Rapid Refresh dataset vs. ERA5 HRES in our study). However, the change in r_{ice} from Caiazzo et al.[18] (+58%) is around two times larger than our study (+26%) because they assumed a larger reduction in the nvPM EI_n (-75% vs. a mean of -52% in our study), where humidity in the contrail plume is distributed to fewer particles. Our estimated change in $\tau_{contrail}$ (-22%) and contrail cirrus cover (-41%) are within range of values compiled from the comparison studies ($\tau_{contrail}$ between -49% and -21%; and contrail cirrus cover between -41% and -15%), and the large range between studies is due to differences in the selected domain area and the assumed reduction in nvPM EI_n (Table S7).

While the change in annual mean contrail cirrus net RF from our study (-44%) appears to be in contrast with Caiazzo et al.[18] (-4% to +18%), these reported values likely represent the mean contrail net RF', i.e., change in radiative flux per contrail area, instead of the annual mean contrail cirrus net RF over a specific domain (refer to Table S1 of Caiazzo et al.[18]). We also note that the range of RF' estimates from Caiazzo et al.[18] (-4% to +18%) was derived from five different assumptions of ice particle habits, while CoCiP provides the weights for a mixture of ice crystal habits as a function of the contrail r_{ice}. Using the definition of RF', the reported values from Caiazzo et al.[18] (-4% to +18%) becomes consistent with the change in net RF' values from this study (-3.9%). Bock & Burkhardt[20] estimated a smaller reduction in the annual mean contrail cirrus net RF from SAF (-15%) relative to our study (-44%), and these differences can likely be attributed to their projected air traffic for 2050: the contrail net RF is known to increase non-linearly with air traffic levels[17,19], and reducing the nvPM EI_n and n_{ice} could have a smaller impact on the contrail climate forcing when air traffic levels are high because the lower ice nucleation rates are offset by the abundance in the total number of contrail ice crystals[20]. Burkhardt et al. estimated a 50% reduction in the annual mean contrail net RF globally for an 80% reduction in nvPM EI_n, while Schumann et al.[21] computed a 39% reduction

in contrail net RF for a 50% reduction in nvPM EI_n, and these values is comparable to our results (-44% in contrail net RF for a 52% reduction in nvPM EI_n).

S4 Targeted use of SAF

Table S8: Statistics on the change in contrail occurrence and annual $EF_{contrail}$ for different strategies where SAF are blended at different ratios and targeted to flights with the largest $EF_{contrail}$ or $\Delta EF_{contrail}$.

Targeted use of SAF	1% Blend	10% Blend	30% Blend	50% Blend	70% Blend	100% Blend
**Target flights with the largest $EF_{contrail}$**						
Number of flights with SAF	477923	43181	13589	7702	5221	3400
% of flights with SAF	100%	9.0%	2.8%	1.6%	1.1%	0.7%
Δ Persistent contrails (km)	92389	131984	124128	119121	114821	109527
Δ Persistent contrails (%)	0.048%	0.069%	0.065%	0.062%	0.060%	0.057%
Δ $EF_{contrail}$ ($\times 10^{18}$ J)	-0.37	-2.88	-4.61	-4.62	-4.14	-3.31
Δ $EF_{contrail}$ (%)	-0.6%	-4.6%	-7.3%	-7.4%	-6.6%	-5.3%
Δ CO_2 EF ($\times 10^{18}$ J)^{a,b}	-0.0508/ -0.4034	-0.0509/ -0.4034	-0.0506/ -0.4032	-0.0505/ -0.4030	-0.0503/ -0.4027	-0.0502/ -0.4024
Δ CO_2 EF (%)^{a,b}	-0.12%/ -0.96%	-0.12%/ -0.96%	-0.12%/ -0.96%	-0.12%/ -0.96%	-0.12%/ -0.96%	-0.12%/ -0.96%
Δ Total EF ($\times 10^{18}$ J)^{b}	-0.42/-0.77	-2.93/-3.28	-4.66/-5.01	-4.67/-5.02	-4.19/-4.55	-3.36/-3.71
Δ Total EF (%)^{b}	-0.40%/ -0.74%	-2.8%/ -3.1%	-4.5%/ -4.8%	-4.5%/ -4.8%	-4.0%/ -4.4%	-3.2%/ -3.5%
**Target flights with the largest $\Delta EF_{contrail}$**						
Number of flights with SAF	477923	45101	14960	9010	6413	4488
% of flights with SAF	100%	9.4%	3.1%	1.9%	1.3%	0.9%
Δ Persistent contrails (km)	92389	129030	125811	124586	125309	123297
Δ Persistent contrails (%)	0.048%	0.067%	0.066%	0.065%	0.065%	0.064%
Δ $EF_{contrail}$ ($\times 10^{18}$ J)	-0.37	-3.27	-5.76	-6.40	-6.32	-5.81
Δ $EF_{contrail}$ (%)	-0.6%	-5.2%	-9.2%	-10.2%	-10.1%	-9.3%
Δ CO_2 EF ($\times 10^{18}$ J)^{a,b}	-0.0508/ -0.4034	-0.0510/ -0.4034	-0.0509/ -0.4035	-0.0510/ -0.4035	-0.0509/ -0.4034	-0.0509/ -0.4034
Δ CO_2 EF (%)^{a,b}	-0.12%/ -0.96%	-0.12%/ -0.96%	-0.12%/ -0.96%	-0.12%/ -0.96%	-0.12%/ -0.96%	-0.12%/ -0.96%
Δ Total EF ($\times 10^{18}$ J)^{b}	-0.42/-0.77	-3.32/-3.67	-5.81/-6.17	-6.45/-6.80	-6.37/-6.73	-5.86/-6.21
Δ Total EF (%)^{b}	-0.40%/ -0.74%	-3.2%/ -3.5%	-5.6%/ -5.9%	-6.2%/ -6.5%	-6.1%/ -6.4%	-5.6%/ -5.9%

[a]: Assumes a 100-year time horizon for CO_2 emissions

[b]: The two values arise from assumptions on the lower and upper bound of the CO_2 lifecycle emissions from SAF.

Table S8 summarises the changes in contrail occurrence and annual contrail energy forcing ($EF_{contrail}$) when the limited supply of SAF is blended at different ratios and targeted to flights

with: (i) the largest $EF_{contrail}$; or (ii) the largest absolute reduction in $EF_{contrail}$ that result from SAF ($\Delta EF_{contrail}$). When SAF is blended at a 50% ratio and targeted to ~1.9% of flights with the largest absolute reduction in $EF_{contrail}$ ($\Delta EF_{contrail}$), the largest reduction in annual $EF_{contrail}$ is achieved (-10.2%). Further increases in the blending ratio beyond 50% would concentrate SAF to a smaller subset of flights (~1.3% of all flights for the scenario with a 70% blend ratio, and ~0.9% of all flights for a 100% blend ratio) and yield a smaller reduction in the annual $EF_{contrail}$ (-10.1% for a 70% blend, and -9.3% for a 100% blend) relative to the distribution with a 50% blend ratio (-10.2%) (Table S8). This phenomenon can be attributed to the non-linearity of ΔnvPM EI_n versus the SAF blending ratio, where further increases to ΔH beyond 0.5% leads to diminishing returns in reducing the nvPM EI_n (Figure S3).

References

1. Brem BT, Durdina L, Siegerist F, et al. Effects of Fuel Aromatic Content on Nonvolatile Particulate Emissions of an In-Production Aircraft Gas Turbine. *Environ Sci Technol*. 2015;49(22):13149-13157. doi:10.1021/ACS.EST.5B04167/SUPPL_FILE/ES5B04167_SI_001.PDF

2. ICAO. Annex 16: Environmental Protection - Volume II - Aircraft Engine Emissions. International Civil Aviation Organization (ICAO). Published 2017. Accessed August 18, 2022. https://store.icao.int/en/annex-16-environmental-protection-volume-ii-aircraft-engine-emissions

3. Moore RH, Thornhill KL, Weinzierl B, et al. Biofuel blending reduces particle emissions from aircraft engines at cruise conditions. *Nature*. 2017;543(7645):411-415. doi:10.1038/nature21420

4. Schripp T, Anderson BE, Bauder U, et al. Aircraft engine particulate matter emissions from sustainable aviation fuels: Results from ground-based measurements during the NASA/DLR campaign ECLIF2/ND-MAX. *Fuel*. 2022;325:124764. doi:10.1016/J.FUEL.2022.124764

5. Bräuer T, Voigt C, Sauer D, et al. Airborne Measurements of Contrail Ice Properties—Dependence on Temperature and Humidity. *Geophys Res Lett*. 2021;48(8):e2020GL092166. doi:10.1029/2020GL092166

6. Voigt C, Kleine J, Sauer D, et al. Cleaner burning aviation fuels can reduce contrail cloudiness. *Commun Earth Environ 2021 21*. 2021;2(1):1-10. doi:10.1038/s43247-021-00174-y

7. Durdina L, Brem BT, Elser M, Schönenberger D, Siegerist F, Anet JG. Reduction of nonvolatile particulate matter emissions of a commercial turbofan engine at the ground level from the use of a sustainable aviation fuel blend. *Environ Sci Technol*. 2021;55(21):14576-14585. doi:10.1021/ACS.EST.1C04744/SUPPL_FILE/ES1C04744_SI_002.XLSX

8. EASA. ICAO Aircraft Engine Emissions Databank (07/2021). Published 2021. Accessed August 17, 2021. https://www.easa.europa.eu/domains/environment/icao-aircraft-engine-emissions-databank

9. Teoh R, Schumann U, Gryspeerdt E, et al. Aviation Contrail Climate Effects in the North Atlantic from 2016-2021. *AtmosChemPhysDiscuss*. Published online 2022. doi:https://doi.org/10.5194/acp-2022-169

10. Cumpsty NA, Heyes A. *Jet Propulsion: A Simple Guide to the Aerodynamics and Thermodynamic Design and Performance of Jet Engines*. Third Edition. Cambridge University Press; 2015. Accessed July 10, 2019.

https://books.google.co.uk/books?hl=en&lr=&id=YVsZCgAAQBAJ&oi=fnd&pg=PR7&dq=cumpsty+and+heyes&ots=b5F45iuQ7t&sig=kEueUVRfAA6sBdB3Hh-yvTrC-jI#v=onepage&q=cumpsty and heyes&f=false

11. Gierens K, Braun-Unkhoff M, Le Clercq P, Plohr M, Schlager H, Wolters F. Condensation trails from biofuels/kerosene blends scoping study. ENER/C2/2013-627. Published 2016. Accessed July 5, 2022. https://ec.europa.eu/energy/sites/ener/files/documents/Contrails-from-biofuels-scoping-study-final-report.pdf

12. Moore RH, Shook M, Beyersdorf A, et al. Influence of jet fuel composition on aircraft engine emissions: A synthesis of aerosol emissions data from the NASA APEX, AAFEX, and ACCESS missions. *Energy & Fuels*. 2015;29(4):2591-2600.

13. Schripp T, Anderson B, Crosbie EC, et al. Impact of Alternative Jet Fuels on Engine Exhaust Composition during the 2015 ECLIF Ground-Based Measurements Campaign. *Environ Sci Technol*. 2018;52(8):4969-4978. doi:10.1021/ACS.EST.7B06244/SUPPL_FILE/ES7B06244_SI_001.PDF

14. Stickles R, Barrett J. *TAPS II Combustor Final Report. CLEEN Program*.; 2013. https://www.faa.gov/about/office_org/headquarters_offices/apl/research/aircraft_technology/cleen/reports/media/TAPS_II_Public_Final_Report.pdf

15. Boies AM, Stettler MEJ, Swanson JJ, et al. Particle emission characteristics of a gas turbine with a double annular combustor. *Aerosol Sci Technol*. 2015;49(9):842-855. doi:10.1080/02786826.2015.1078452

16. Schumann U. A contrail cirrus prediction model. *Geosci Model Dev*. 2012;5(3):543-580. doi:10.5194/gmd-5-543-2012

17. Kärcher B. Formation and radiative forcing of contrail cirrus. *Nat Commun*. 2018;9(1):1824. doi:10.1038/s41467-018-04068-0

18. Caiazzo F, Agarwal A, Speth RL, Barrett SRH. Impact of biofuels on contrail warming. *Environ Res Lett*. 2017;12(11):114013. doi:https://doi.org/10.1088/1748-9326/aa893b

19. Burkhardt U, Bock L, Bier A. Mitigating the contrail cirrus climate impact by reducing aircraft soot number emissions. *npj Clim Atmos Sci*. 2018;1(37):1-7. doi:10.1038/s41612-018-0046-4

20. Bock L, Burkhardt U. Contrail cirrus radiative forcing for future air traffic. *Atmos Chem Phys*.

2019;19(12):8163-8174. doi:10.5194/acp-19-8163-2019

21. Schumann U, Jeßberger P, Voigt C. Contrail ice particles in aircraft wakes and their climatic importance. *Geophys Res Lett*. 2013;40(11):2867-2872. doi:10.1002/grl.50539
